# Advances in Regenerative Therapies for Inflammatory Arthritis: Exploring the Potential of Mesenchymal Stem Cells and Extracellular Vesicles

**DOI:** 10.3390/ijms26125766

**Published:** 2025-06-16

**Authors:** Timofey O. Klyucherev, Maria A. Peshkova, Maria D. Yurkanova, Nastasia V. Kosheleva, Andrey A. Svistunov, Xing-Jie Liang, Peter S. Timashev

**Affiliations:** 1 Institute for Regenerative Medicine, I. M. Sechenov First Moscow State Medical University, 119991 Moscow, Russia; 2 Sechenov First Moscow State Medical University, 119991 Moscow, Russia; 3 CAS Key Laboratory for Biomedical Effects of Nanomaterials and Nanosafety, CAS Center for Excellence in Nanoscience, National Center for Nanoscience and Technology of China, Beijing 100190, China; 4 University of Chinese Academy of Sciences, Beijing 100049, China

**Keywords:** extracellular vesicles, exosomes, mesenchymal stem cells, osteoarthritis, rheumatoid arthritis, inflammation

## Abstract

Inflammatory arthritis, including rheumatoid arthritis (RA) and osteoarthritis (OA), is a group of degenerative joint diseases that result in reduced mobility and a prevalent cause of disability. Despite differing etiologies, both conditions involve inflammation, affecting only the joints in OA and systemic in RA due to its autoimmune nature. Regenerative medicine offers promising alternatives, with a focus on the therapy with mesenchymal stem cell (MSC) and their secreted extracellular vesicles (EVs). MSC-derived EVs have shown great potential in modulating inflammatory pathways and promoting tissue repair in the preclinical models of RA and OA. Additionally, EVs from immune cells exhibit strong anti-inflammatory effects, reducing cartilage and bone degeneration. This review highlights the recent progress in MSC-based and EV-based therapies for OA and RA, discussing the bioengineering approaches that enhance the therapeutic efficacy, stability, and targeting of EV. It also addresses the major challenges in translating EV therapy from the laboratory to clinical practice and discusses strategies to overcome these obstacles in the treatment of inflammatory arthritis.

## 1. Introduction

Rheumatoid arthritis (RA) and osteoarthritis (OA) are heterogeneous diseases in terms of their etiology and mechanisms of development, leading to significant deterioration in the quality of life and being common causes of disability [1,2]. RA is a chronic systemic autoimmune inflammatory disease that causes damage to the joints, connective tissue, muscles, tendons, and fibrous tissue. The global prevalence of RA is about five cases per 1000 adults [3,4]. OA is a widespread, progressive, and degenerative joint disease characterized by degenerative processes in the articular cartilage, subchondral bone, and inflammation of the synovial membrane, where factors such as age, genetic predisposition, gender, lifestyle, and joint injuries contribute to the joint destruction and disease progression [5]. The cost of OA therapy in the USA amounts to $100 billion, and since aging is one of the main risk factors for the disease, its prevalence will only increase, creating a heavy burden on the national healthcare systems [6].

The etiology of both types of arthritis is not fully understood. In RA, the MHC Human leukocyte antigen (HLA)-DRB1 gene is considered the main risk factor, as well as a high level of autoantibodies to citrullinated protein found in 80% of patients [7,8,9]. In the case of OA, the risk factors may include disturbances in the chondrocyte metabolism and synthesis and degradation of the extracellular matrix (ECM), inflammation due to aging, or mechanical stress [10]. Secondary OA often develops after injuries or infections and is characteristic of younger patients [11,12].

One of the primary triggering factors of OA is the alteration of the chondrocyte metabolism in response to mechanical damage and aging [13]. Chondrocytes are a population of cells with limited regenerative capacity. They play a crucial role in maintaining the integrity of the ECM [14]. In response to pathological factors, chondrocytes tend to undergo hypertrophy and secrete catabolic factors, including Runt domain factor-2, collagen 2, and various metalloproteinases (MMP) [15]. The cartilage degradation during the OA progression is accompanied by the release of damage-associated molecular patterns (DAMP), which activate synovial fibroblasts and macrophages [16]. This activation promotes the pro-inflammatory state characterized by the secretion of inflammatory cytokines, including tumor necrosis factor (TNF)-α, interleukin (IL)-1, IL-6, and MMP (MMP-1, MMP-3, and MMP-13) The secretion of these inflammatory factors and proteases creates an inflammatory microenvironment within the knee joint, leading to further cartilage and subchondral bone damage [16]. This, in turn, exacerbates the clinical manifestations of OA, such as joint dysfunction [17].

**Figure 1:**
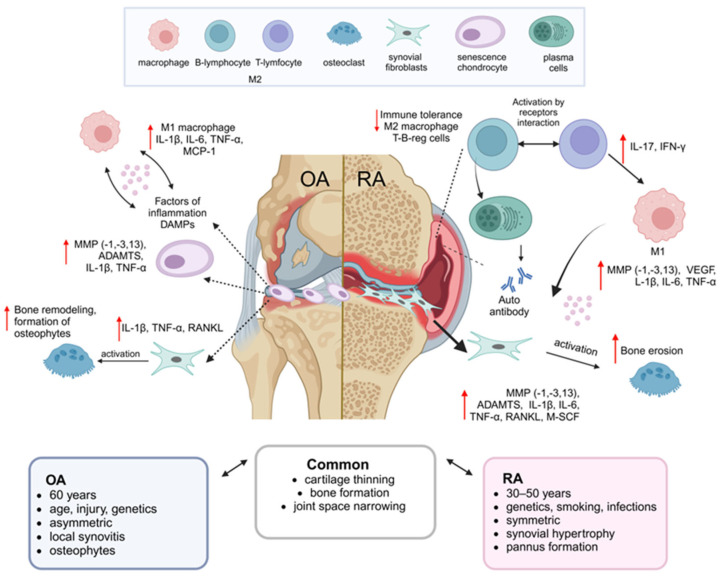
Comparison of the main features of OA and RA. The figure presents a brief characterization of the common and specific features of each disease, including differences in etiology, clinical manifestations, and pathogenic mechanisms for OA and RA [1,26,27,28].

In the RA progression, the synovial membrane thickening and activation of two synoviocyte populations—macrophage-like synoviocytes (MLS) and fibroblast-like synoviocytes (FLS)—can be observed. Both types of synoviocytes secrete chemokines and inflammatory cytokines, such as IL-1, IL-6, TNF-α, prostaglandins, and leukotrienes [18,19]. Additionally, FLS produces significant amounts of MMP, contributing to ECM degradation. These secreted factors stimulate synovial infiltration by macrophages and CD4+ T-cells (Th1 and Th17), which secrete TNF-α, IFN-γ, IL-6, IL-1β, and IL-17 [20]. B-cells secrete autoantibodies, such as rheumatoid factor, and antibodies against citrullinated proteins [21,22]. The increased TNF-α secretion enhances B-cell expression of the nuclear factor kappa-light-chain-enhancer of activated B cells (NF-κB) ligand (RANKL), promoting osteoclastogenesis and leading to increased bone resorption [23,24]. The infiltration of immune cells, along with the proliferation and activation of synoviocytes, results in the formation of RA-specific pannus, which erodes the cartilage and bone during the later stages of RA pathogenesis [25]. The common properties, as well as the differences in the pathogenesis of OA and RA, are presented in Figure 1.

Modern recommendations for the treatment of RA include symptomatic treatment and disease-modifying drug therapy, whereas OA therapy is primarily aimed at symptom relief [29,30]. RA therapy includes disease-modifying antirheumatic drugs (DMARDs), nonsteroidal anti-inflammatory drugs (NSAIDs), and glucocorticoids, targeting inflammation and symptoms [31,32]. Although synthetic and biological DMARDs are effective, they are expensive and have significant side effects [1,33]. There are no drugs available for OA that affect the pathogenesis of the disease; pharmacotherapy aims at symptom relief using NSAIDs, glucocorticoids, hyaluronic acid injections, and platelet-rich plasma [29,34,35]. In severe cases, joint replacement with an endoprosthesis is required, which carries high risks [34,36].

The need for effective therapy for RA and OA with an adequate safety profile has led to the investigation of cell therapy based on mesenchymal stem cells (MSC) and components of their secretome—extracellular vesicles (EVs), which have shown efficacy due to their immunosuppressive and regenerative effects [37,38,39]. This review will compile the studies on MSC and MSC-EV therapy for OA and RA, as well as therapies based on other cell sources. In addition to unmodified EV, attention will be given to various approaches to modify MSC and EV by altering cultivation conditions, genetic modifications, and creating combined tissue engineering constructs that incorporate scaffolds and therapeutic components consisting of EV or MSC. The prospects and challenges of transitioning EV therapy for OA and RA from bench to bedside will also be discussed. This manuscript presents a narrative review based on the literature retrieved from bibliographic and abstract databases, including Scopus, PubMed, Google Scholar, and ClinicalTrials.gov.

## 2. Cell Therapy

MSCs have garnered significant attention from scientists and clinicians in the context of treating many diseases, including OA and RA, due to their multimodal regenerative and immunomodulatory properties [40,41]. MSCs are non-hematopoietic spindle-shaped cells that adhere to plastic and possess the ability to self-renew and differentiate into the chondrogenic, osteogenic, and adipogenic lineages [42,43]. Until recently, the human or animal bone marrow has been the main source of material for preclinical studies and clinical trials. Adult stromal cells are found in most tissues and organs, and the following sources have been used to treat knee osteoarthritis: bone marrow, peripheral blood, adipose tissue, trabecular bone, synovial fluid, and synovial membrane [44,45,46,47,48]. In a recent systematic review and meta-analysis including 15 studies, Chen et al. showed that MSC transplantation effectively treats patients with OA, with autologous from bone marrow mesenchymal stem cells (BMMSC) potentially providing more benefit [49]. The isolation of mesenchymal stromal cells from adipose tissue is recognized as a potential way to obtain a large number of autologous cells for the cell therapy of osteoarthritis due to their relatively easy harvesting and chondrogenic potential. In addition, these cells are less susceptible to aging and are independent of the patient’s physiologic state, gender, and age [50,51]. According to the International Society for Cellular Therapy, MSCs are characterized by the expression of the surface markers cluster of differentiation (CD)73, CD105, and CD90, while lacking the expression of CD34, CD14, CD45, and CD11b [42,52,53]. The studies on the therapeutic properties of MSC for the treatment of inflammatory and degenerative diseases have shown that MSCs exert anti-inflammatory effects and stimulate regeneration through paracrine effects and cell–cell interactions [54,55,56,57].

### 2.1. The Immune-Modulating Effect

In inflammatory diseases of the musculoskeletal system, there is a disruption of balance in the innate and regulatory immune responses, characterized by an increase in the proportion of pro-inflammatory M1 macrophages and Th1 and Th17 T-cells, with a decrease in cells with anti-inflammatory and regulatory phenotypes, such as M2 macrophages and T-reg cells [58,59,60].

The immunosuppressive effect of MSC can be mediated through cell–cell contact with T-cells and natural killer (NK) cells by interacting with the MSC-expressed surface molecule HLA-G, a non-classical MHC class I molecule that can suppress the action of activating NK receptors [61,62]. Another mechanism for the receptor regulation of T-cell activity by MSC is mediated through intercellular adhesion molecules ICAM-1 and ICAM-2, as well as the surface ligand for programmed death-1 (PD-1), which allows the inhibition of allogeneic differentiation of T-helper (Th)17 cells [63,64]. Furthermore, MSC inhibits phosphorylation of extracellular signal-regulated kinases (ERK)1/2 and causes the activation of mitogen-activated protein kinase p38 (MAPK) in B cells, leading to cell cycle arrest in the G0/G1 phase [65].

Moreover, the immunosuppressive effect of MSCs is caused by the paracrine mechanism. MSCs enhance the immune tolerance of Tregs and tolerogenic dendritic cells (tDCs) by inhibiting T-cell proliferation through MSC-secreted indolamine-2,3-dioxygenase (IDO), which is stimulated by the formation of kynurenine from tryptophan, necessary for T-cell proliferation [66,67]. Prostaglandin E2 (PGE2) produced by MSC suppresses the IFN-γ production in NK cells and activates CD4+ T cells, resulting in the attenuation of inflammation induced by Th1 cells [68]. In RA and OA, there is a disruption in the balance of osteoblast and osteoclast activity, leading to bone destruction and resorption [69]. Increased osteoclastogenesis is influenced by M1 macrophages, which secrete TNF-α and IL-1β; these inflammatory cytokines activate synovial fibroblasts that secrete macrophage colony-stimulating factor (M-CSF) and RANKL, both necessary for the survival and maturation of osteoclasts [70,71]. Different types of MSC can alleviate the pro-inflammatory environment in joints in OA and RA by inducing polarization of macrophages into the M2 phenotype in vitro, as well as inhibiting inflammatory activation of synovial macrophages in vivo [72,73,74]. Thus, through the production of TNFα-stimulated gene/protein 6, PGE2, and IDO, MSC attenuates the inflammatory process in inflammatory arthritis by reducing M1 macrophage polarization, which is associated with the secretion of TNF-α and IL-1, and promoting M2 polarization [75]. These changes occur due to the release of TSG-6 in response to TNF-α produced by activated M1 macrophages. TSG-6 interacts with CD44 on macrophages, reducing Toll-like receptor (TLR)2/NF-κB signaling and consequently decreasing the secretion of inflammatory mediators (nitric oxide, TNF-α, and IL-1) [75,76]. The effects of TSG-6 have been replicated in an in vivo model of dextran sulfate sodium-induced colitis. This factor, secreted by adipose-derived MSC, alleviates inflammation upon intraperitoneal injection by promoting phenotypic switching of macrophages toward the M2 phenotype [77]. In turn, MSC-derived PGE2 binds to EP2 and EP4 receptors on macrophages and promotes the production of immunosuppressive IL-10 [78]. MSC therapy application in RA has shown that MSC can suppress the differentiation and maturation of mature osteoclasts through MSC-secreted osteoprotegerin, as well as via suppressing the RANKL-induced osteoclastogenesis, thus reducing the progression of bone tissue erosion [79].

Among the paracrine factors secreted by MSC that promote regenerative processes in OA and RA, the purinergic signaling molecule adenosine plays a pivotal role. Adenosine is produced via the enzymatic activity of CD39 and CD73 (ecto-5′-nucleotidase), both of which are expressed on MSC [80,81]. This mechanism modulates inflammatory and apoptotic responses in chondrocytes, resulting in reduced inflammation and catabolism, and decreased secretion of IL-6, IL-1β, TNF-α, and PGE2, ultimately mitigating cartilage degeneration in OA and RA [82,83]. Adenosine and CD73 expression also partially mediate the immunosuppressive and proliferative effects of MSC on human macrophages and T lymphocytes. Suppression of CD73 or adenosine A2A receptor expression negatively impacts the immunosuppressive activity of MSC, underscoring the critical role of CD73 in modulating MSC immunoregulatory function [84,85]. MSC-mediated regulation of adenosine synthesis via CD39/CD73 also modulates osteoclastogenesis in autoimmune arthritis, such as RA [86,87]. In this context, gingiva-derived MSC inhibits the NF-κB and p65/p50 expression in vitro and reduces the RANKL expression in the synovial tissue and osteoclast formation in an in vivo model of autoimmune arthritis in mice [86,87].

The immunomodulatory properties and differentiation potential of MSC can be improved through various approaches. The key approaches include modulation of cultivation conditions, such as priming with inflammatory cytokines, and cultivation under hypoxia [78,88,89]. MSCs are quite sensitive to oxygen fluctuations, particularly to high concentrations that induce oxidative stress in MSC, and they show the highest viability when cultured under reduced oxygen levels [90,91]. Several studies have shown that cultivation under hypoxia enhances the regenerative potential of MSC by increasing Hypoxia-inducible factor-1α (HIF-1α)-dependent autophagy [92]. The ability to enhance the immunosuppressive effect in treating diseases associated with inflammation and immune dysregulation has attracted wide attention from researchers. Cultivation of MSC in the presence of inflammatory cytokines or various growth factors, such as TGF-β and bone morphogenetic proteins (BMP), can enhance the immunomodulatory properties of MSC. For example, priming MSC in the presence of IFN-γ can inhibit the effector functions of T-cells, as well as enhance the polarization of monocytes into the M2 macrophage phenotype, secreting IL-10, which leads to a reduction in the number of Th17 cells [78,93,94]. Moreover, calcium channels regulated by physical stimuli also play an important role in the chondrogenic differentiation of MSC [95]. These channels increase the concentration of intracellular calcium, which leads to the triggering of signaling pathways that induce the expression of cartilage-specific genes and promote the synthesis of cartilage-specific proteins (type 2 collagen, type 9 collagen, aggrecan, and cartilage oligomeric matrix protein) during chondrogenic differentiation [95]. Additional application of various physical stimuli—mechanical, electric, electromagnetic, or magnetic fields—can lead to an increase in the calcium concentration and, ultimately, to the activation of chondrogenic gene expression in MSC [96,97]

### 2.2. ECM Synthesis

The progressive destruction of the cartilage and bone tissue caused by an imbalance of the ECM synthesis and degradation is one of the main negative manifestations of both OA and RA [98,99]. The primary role in the degradation of the ECM in the cartilage, consisting mainly of different types of collagens and proteoglycans, is played by MMPs, whose increased expression predominates over the levels of cartilage anabolic markers such as type II collagen [100,101]. MSC therapy can stimulate the regenerative processes in the cartilage tissue by reducing the expression of the ECM catabolism factors such as MMP13 and ADAMTS-5 disintegrins, while increasing the content of tissue inhibitors of MMPs [102,103]. In addition, Wang et al. showed the feasibility of MSC application in the therapy of degenerative joint diseases, as they found an increase in Col 2α1 gene expression in the knee joint cartilage, which encodes the α-1 chain of collagen II, after MSC-based therapy [104].

### 2.3. Chondrogenesis

The significant component of the pathogenesis of osteoarthritis is a decrease in chondrocyte proliferation, their ability to autophagy, and increased apoptosis [105]. The chondrogenesis is primarily caused by the chondrogenic differentiation of MSC triggered by some growth factors or increased activity of chondrocyte progenitors and chondrocytes stimulated by MSC [106]. Thus, UCMSC demonstrated high chondrogenic potential confirmed by the increased expression of the sex-determining region of the Y-chromosome-transcription factor 9 (Sox9) as a marker of chondrocyte precursors [107]. BMP is a subfamily of the TGF-β superfamily responsible for the induction of bone and cartilage formation. Increased levels of BMP6 promoted the chondrogenesis of mesenchymal stem cells [108]. Overexpression of key transcription factors to maintain pluripotency and self-renewal, such as Nanog and Oct4, also enhanced the chondrogenic properties of cells [109]. Zhang et al. found that intra-articular injection of MSC from the human umbilical cord blood reduced the development of joint lesions due to the increased expression of collagen and ki67 in the articular cartilage [103].

The oxidative stress in human chondrocytes can cause DNA damage and promote cell aging [110]. Reactive oxygen species (ROS) are significant in the signaling pathways activated by IL-1β in chondrocytes [111]. Platas et al. investigated the effect of the ROS on the modification of of adipose tissue MSC (ATMSC)-dependent proteins. In chondrocytes derived from an osteoarthritis model, IL-1β rapidly induced ROS synthesis and increased the level of 4-hydroxy-2-nominally modified proteins, while ATMSC promoted a decrease in their concentration [112]. Aging and stress affect the phenotype of chondrocytes in osteoarthritis, while age-related mitochondrial dysfunction and associated oxidative stress may contribute to chondrocyte aging [113]. Studies have shown that mitochondria are transferred from bone marrow mesenchymal stem cells (BMMSC) to chondrocytes in osteoarthritis. One study demonstrated that co-culture with mitochondria of MSC increased the mitochondrial membrane potential compared to chondrocytes that did not receive mitochondria. Moreover, the activity of mitochondrial respiratory chain enzymes and the level of adenosine triphosphates significantly increased [114].

### 2.4. Tissue Engineering and MSC

Tissue engineering is a rapidly developing technology. These approaches enhance the regenerative potential of MSC by creating the 3D conditions that mimic the natural MSC niche formed by the ECM, as cultivation in the 2D conditions makes the cell phenotype more pronouncedly different from the in vivo conditions, limiting the therapeutic potential of MSC-based cell therapy [115,116]. The scaffold materials for encapsulating MSC can include both synthetic and natural polymers, such as biopolymers: fibrin, collagen hydrogel, silk, chitosan, and various synthetic polymers, enhancing MSC survival and their ability to undergo chondrogenic and osteogenic differentiation [117,118,119,120]. Combining MSC with a hydrogel reduces the cell component loss during the implantation procedure by decreasing the mechanical stress [121]. The hydrogel’s ability to retain MSC at the injection site, along with creating a favorable environment for cell growth and differentiation, results in a longer therapeutic effect of MSC therapy than administering only the cell component, contributing to the preservation of the therapeutic effect in OA patients for up to 3 years [122]. In addition, Gonzalez-Fernandez et al. covalently linked glucose molecules to hyaluronic acid and used the resulting molecule as a base for the creation of an MSC-enriched hydrogel [123]. During in vitro modeling experiments, it was found that the addition of glucose to the hydrogel increased the viability of MSC by 71% [123]. The authors explain this result by the presence of β-glucosidase in the joint cavity, which hydrolyzes glycosidic bonds between glucose and hyaluronic acid molecules, resulting in the release of glucose molecules that provide MSC with energy [123].

Due to their high therapeutic potential, MSCs have shown their therapeutic efficacy in preclinical settings, providing significant therapeutic effects in treating osteoarticular diseases such as RA and OA. However, despite the high therapeutic potential, MSC-based cell therapy itself raises several safety concerns. MSC may exhibit undesirable proliferative and differentiation capabilities and risk of phenotype loss, with the greatest concern being the risk of tumor transformation [124,125]. Another undesirable factor that can cause an excessive immune response during allogeneic MSC transplantation in vivo is the expression of the MSC molecule MHC I, which can trigger an undesirable immune response in the patient [126]. However, the question of whether MSC provokes the host immune rejection upon allogeneic transplantation remains under debate. This is due to the fact that MSC does not express HLA-DR or the co-stimulatory molecules CD80, CD86, and CD40, which are typically required for the activation of T and B lymphocytes [127,128]. In general, in the absence of a pro-inflammatory microenvironment, MSC exhibit immunosuppressive activity toward macrophages, NK cells, T cells, and B cells, and do not typically induce severe immune reactions following in vivo transplantation [129,130]. Therefore, from a safety standpoint, therapy by extracellular vesicles from MSC and other cellular sources looks more promising for OA and RA therapy; research on this topic will be discussed in the following sections.

## 3. Therapy of OA and RA with Unmodified EV

One of the promising analogs of cellular therapy for inflammatory joint diseases is the use of extracellular vesicles (EVs) [131]. EVs have heterogeneous origins and functions within the body; they include exosomes, microvesicles, and apoptotic bodies [132,133]. Exosomes are nanoscopic particles of endosomal origin with an average size of 30–150 nm [134,135]. Exosomes are formed by the invagination of clathrin-coated plasma membrane microdomains, leading to the formation of multivesicular bodies. During exosome biogenesis, they fuse with the plasma membrane, followed by the secretion of exosomes into the intercellular space [136,137]. Exosomes can be differentiated from other types of EV using the endosomal markers CD9, CD63, CD81, TSG101, and Alix [138]. According to the MISEV2023 guidelines, it is recommended to use the general term “EV” instead of “exosomes” or “microvesicles,” unless the biogenetic pathway has been clearly demonstrated and selective isolation methods have been employed [135]. In this narrative review, specific terms such as “exosomes” are used only in those studies where the authors explicitly identify the EV subtype and provide the characterization of specific EV markers. Apoptotic bodies are relatively large EVs consisting of subcellular fragments (average size from 1 to 5 µm); they are formed during apoptotic cell death [139,140]. Microvesicles can be larger than exosomes with an average size of 100–1000 nm, although their sizes may overlap with the size of exosomes [141]. This EV subtype is released by the budding of the plasma membrane and participates in intercellular communication through the local or paracrine transmission of signaling molecules [139,141].

Currently, the increasing evidence highlights the role of EV in the progression of diseases associated with the degenerative processes in the cartilage tissue, such as OA and RA. [142,143]. EVs associated with the pathogenesis of RA and OA differ in their composition compared to those from healthy patients and tend to accumulate in the bloodstream and synovial fluid of individuals suffering from these diseases [144,145,146]. Studying the role of endogenous EV in OA and RA development contributes to a deeper understanding of the intricate mechanisms underlying these multifactorial pathologies and allows for the consideration of these microparticles as potential biomarkers for disease diagnosis [147]. In OA patients, exosomes circulating in the synovial fluid carry elevated levels of inflammatory cytokines IL-1β and TNF-α [145]. The inflammatory mediators transported by EV contribute to the activation of FLS, promoting cartilage ECM degradation and the formation of an inflammatory microenvironment [144]. The pathological calcification of the cartilage tissue is considered by researchers as a critical factor in the early pathogenesis of OA [143]. Calcified EV formed in autophagosomes, containing autophagy-derived microtubule-associated proteins 1A/1B light chain 3B positive EV, can initiate pathological cartilage calcification. Regulating the secretion of these EV is regarded as a promising therapeutic target for OA [143]. Senescent chondrocytes are a source of EV that negatively affect the ECM deposition and accelerate the aging of surrounding cells. The removal of senescent chondrocytes has been shown to favorably influence the composition of EV, slowing degenerative changes in the cartilage tissue [148]. Subchondral osteoblasts secrete exosomes that disrupt chondrocyte metabolism, enhancing the expression of catabolic-related genes [149]. In RA patients, the IgM rheumatoid factor has been detected in plasma EV, and its presence in circulating exosomes has been associated with more severe disease progression [150]. EV from RA patients has been shown to stimulate the M1 polarization of macrophages with a pro-inflammatory phenotype and to support the survival of T and B lymphocytes when co-cultured with these microparticles, modulating the cellular behavior characteristic of the RA development in vivo [151]. Additionally, the evidence suggests that exosomes negatively affect T-cell regulation by disrupting the Th17/Treg balance and promoting increased levels of inflammatory cytokines [142]. Exosomes from the synovial fluid of RA patients stimulate osteoclastogenesis, and these exosomes can be detected in RA patients but not in OA patients. This specificity suggests that exosomes could serve as diagnostic biomarkers for RA [152]. The invasive synovial tissue phenotype is one of the key attributes of RA pathogenesis [153]. In a study by Frank-Bertoncelj M et al., it was discovered that exosomes in RA patients contained Toll-like receptor 3 and polyinosinic-polycytidylic acid. When these EVs interact with synovial fibroblasts, they induce the pathological phenotype characteristic of RA [154]. Regulating the biogenesis and cargo of EV represents a promising research direction for identifying the potential therapeutic targets for the OA and RA treatment.

To date, an increasing number of studies are exploring the potential use of EV-based cell-free therapies for modulating the inflammatory and dystrophic processes that occur due to joint inflammatory diseases in OA and RA [155,156,157,158]. The application of EVs derived from MSCs has shown that the effect of EV is not inferior in its effectiveness to MSC-based cellular therapy [159,160]. MSC-derived EVs, unlike MSCs, are safer due to their lower immunogenicity and lower likelihood of a nonspecific interaction with circulating proteins [157,161,162]. It should be noted, however, that similar to their parent cells (e.g., MSCs), exosomes derived from these sources may contain small amounts of MHC-I molecules on their surface [163]. Nevertheless, proteomic analyses have shown that MSC-derived exosomes do not contain significant levels of MHC-I. Moreover, these exosomes predominantly exert immunosuppressive effects and do not provoke strong immune responses in vivo [163,164]. One of the significant risks of MSC-based cellular therapy is their risk of abnormal differentiation and tumor transformation, while EVs do not pose such risks since they are not capable of replication [165]. EVs protect their cargo of biologically active molecules, proteins, and nucleic acids and are also capable of crossing biological barriers [166,167]. The ability of EVs to interact specifically with target cells, along with their good safety profile, makes EVs a more optimal delivery tool for both endogenous molecules that regulate cellular processes and drug molecules loaded into EVs [168].

One of the key advantages of EV therapy for degenerative joint diseases is the management of autoimmune and inflammatory processes at both the systemic and local tissue microenvironment levels, which helps to alleviate pain and inflammation and prevent destructive processes in the joints [165,169]. Excessive inflammation in RA arises from the imbalance between regulatory T cells (Treg) and Th1/Th17, with the latter producing inflammatory cytokines such as IFN-γ and IL-17, enhancing migration and activation of FLS, granulocytes, and macrophages in the knee joint [170,171,172,173]. Gingival MSC (GMSC) exosomes exert their therapeutic effect by inhibiting the IL-17RA-Act1-TRAF6-NF-κB signaling pathway, reducing the secretion of inflammatory cytokines TNF, IL-6, and IL-1β [157]. The impact on the IL-17 cytokine secretion is one of the important directions in modifying the inflammatory process in RA, as this cytokine, through the activation of IL-17RA, transmits a signal via the Act1-TRAF6 pathway, leading to the activation of NF-κB, MAPK, and PI3K pathways [157,174]. EV from umbilical cord mesenchymal stem cells (UCMSC) are able to change the Treg/Th17 ratio by increasing the proportion of Treg in RA, while they increase the secretion of TGF-β in the blood serum, which leads to the inhibition of pathomorphological changes in the synovial tissue [175]. The immunosuppressive effect of exosomes derived from BMMSC contributed to a better therapeutic effect compared with microparticles and parent cells of MSC, effectively reducing inflammation, which was accompanied by an increase in the number of regulatory B-cells expressing IL-10 in lymph nodes [176].

Destructive processes in the cartilage, bone, and tendons are among the main signs of progression of both OA and RA [99,177]. A characteristic sign of RA is changes in the synovial membrane, which are accompanied by excessive proliferation of FLS and immune cell infiltration, leading to the formation of synovial hyperplasia [178]. Increased activation of FLS and immune cells such as lymphocytes and macrophages enhances the expression and secretion of inflammatory cytokines, growth factors, and adhesion molecules TNF-α, IL-6, IL-1β, VEGF, CAM-1, VCAM-1, creating a pro-inflammatory microenvironment in the synovial membrane in RA, which contributes to the cartilage tissue resorption and increased activation of neovascularization [179,180,181]. One of the main destructive factors for both OA and RA is the increased production of proteases MMP by metabolically active chondrocytes and FLS, induced by pro-inflammatory cytokines IL-1β and TNF-α, which degrade the ECM components of hyaline cartilage such as MMP-1, -3, and -13 [182,183]. The action of BMMSC)-exosomes can mitigate the effects of inflammatory cytokines, such as TNF-α and IL-6, as well as proteases MMP1 and MMP13 in FLS treated with IL-1β, with one mechanism of this effect being the suppression of the NF-κB signaling pathway, leading to the reduced expression of these pathological factors [184,185]. Suppression of NF-κB signaling is relevant in both OA and RA, as there are cross-effects between the increased intracellular signaling via NF-κB, leading to the induction of MMPs, whose excessive secretion creates a catabolic microenvironment associated with more pronounced destruction of the ECM components in the cartilage tissue [186,187]. Exosomes secreted by MSCs contain active CD73 and may mediate the paracrine immunosuppressive activity of MSCs toward macrophages by promoting M2 polarization. These exosomes enhance the adenosine production, which, through the activation of adenosine A2A and A2B receptors, induces phenotypic changes in macrophages via AKT/ERK-dependent signaling pathways, leading to the development of the M2 phenotype characterized by the increased expression of M2-associated markers such as arginase-1, IL-10, IL-1RN, and CD206 [188]. Small EV from adipocyte MSC (ADMSC) can improve the inflammatory and catabolic environment in chondrocytes and synoviocytes by suppressing the secretion of pro-inflammatory cytokines IL-1β, IL-6, IL-8, and monocyte chemoattractant protein 1 (MCP-1) in both cell types by suppressing NF-κB, whose expression was induced by IL-1β [155].

There is a correlation between the increased production of ROS, oxidative stress, and DNA damage in chondrocytes and the synovial membrane, which induces cellular apoptosis. Therefore, managing oxidative stress represents a promising therapeutic target for arthritis [110,189]. Exosomes from human UCMSC affect ROS production, which causes damage to chondrocytes, alleviating oxidative stress through the suppression of exosomal miR-100-5p, which inhibits the NOX4 expression [190]. Exosomes from UCMSC can suppress inflammation by reducing the levels of Nod-like receptor family pyrin domain containing 3 (NLRP3) in macrophages through the inhibition of METTL3 via miR-1208, leading to decreased production of inflammatory cytokines [191]. EVs from MSCs provide a high therapeutic potential for the treatment of inflammatory and degenerative joint diseases, preserving the therapeutic effects of EV-producing cells while ensuring a better safety profile. This allows for the consideration of unmodified EVs as an alternative cell-free platform for delivering paracrine factors of stem cells to the damaged joint area, providing a therapeutic effect locally.

In addition to exosomes and microvesicles from MSCs, the components of the ECM of mucosal soft tissues have been identified relatively recently as donors of EV with immunomodulatory properties, termed matrix-bound nanovesicles (MBVs) [192]. MBVs obtained from the ECM UCMSC have a significantly different composition from exosomes in the proteome and are enriched with clusters of leukocyte activation, cell migration, and ECM component formation [193]. MBV carries miRNA125b-5p, 143-3p, and 145-5p and can manage the inflammatory process by altering the macrophage polarization, promoting the increase in M2 macrophage populations with anti-inflammatory and immunoregulatory profiles [194]. In the treatment of acute and chronic RA, MBV at the level of standard methotrexate therapy contributed to better bone tissue remodeling in mice and increased the population of M2-like macrophage phenotypes [195].

In addition to MSCs, EVs (exosomes, microvesicles, apoptotic bodies) derived from immune cells, including M2 polarized macrophages, neutrophils, and granulocytic myeloid-derived suppressor cells (G-MDSC), can regulate the inflammatory process in RA and OA [196,197,198,199]. Controlling the macrophage phenotypes is one of the therapeutic targets for OA and RA, and EVs derived from M2 macrophages can facilitate the reprogramming of macrophages in situ by increasing the population of M2 macrophages with an anti-inflammatory profile in the synovial tissue in RA [196]. Exosomes derived from monocytes differentiated into IL-4 M2 macrophages can stimulate the differentiation and ECM production by chondrocytes through the transfer of mRNA and Sox9 protein, which ensures the differentiation of chondrocytes from precursor cells [199,200]. In addition to exosomes, apoptotic bodies have shown outstanding therapeutic potential in OA. Apoptotic bodies from M2 macrophages enriched in miR-21-5p, unlike pro-inflammatory M1 macrophages, can modulate the macrophage phenotype in the synovial tissue, reducing inflammatory cytokines IL-1β, IL-6, TNF-α, IFN-γ, and preserving the cartilage tissue structure in OA [201].

Microvesicles from neutrophils can positively affect the cartilage degradation in RA by influencing chondrocyte homeostasis, preventing their apoptosis by reducing the secretion of prostaglandin E2 and IL-8, as well as by reducing the inflammatory activation of FLS and M1 macrophages [198,202]. Exosomes from G-MDSC have immunomodulatory properties in RA through their diverse exosomal cargo of miR-29a-3p and miR-93-5p nucleic acids [158,197]. The presence of miR-29a-3p and miR-93-5p in G-MDSC exosomes can influence another aspect of the RA autoimmunity by reducing the elevated Th1 and Th17 levels in RA, which is accompanied by a decrease in inflammatory cytokines IFN-γ and IL-17A [197]. Immune cells can be an effective alternative source of EV with tropism to cells with high phagocytic activity, such as resident macrophages, making them a promising therapeutic agent for cell modification in OA and RA [203]. Detailed studies are needed to compare EVs from various sources in terms of safety and therapeutic efficacy. However, there are several challenges to advancing therapy based on unmodified EVs that need to be considered. First, there are risks of high variability in the EV cargo content since cultured cells can undergo aging and exposure to external cultivation factors. There are also concerns regarding the specificity and targeting of therapeutic EVs to target cells. These issues highlight the need for approaches that ensure the modification of EV-producing cells and the EVs themselves to preserve the signaling molecules with therapeutic potential within EVs for OA and RA, as well as surface modification of EVs using various technologies to ensure specific interactions with target cells.

## 4. Modified Extracellular Vesicles

Although extracellular vesicles from unmodified parent cells demonstrate the therapeutic efficacy in in vitro and in vivo models of OA and RA, the application of bioengineering approaches can enhance the therapeutic efficacy, improve the pharmacokinetic properties, increase the EV stability, and increase their ability to target specific cells [204,205]. The existing EV modification methods can be divided into approaches applied before the EV isolation, targeting EV-producing cells through genetic manipulation and alteration of cultivation conditions, as well as post-isolation approaches involving the addition of therapeutic molecules to EV or surface modification of EV using various chemical methods [206,207,208]. Indirect EV modification approaches include modifying the cultivation conditions, culturing cells under hypoxia, and conditioning cells with various inflammatory cytokines, antioxidants, and growth factors [209,210]. Additionally, culturing cells in 3D conditions can significantly improve the therapeutic efficacy of EV [211].

### 4.1. Hypoxic Method

Culturing under low oxygen conditions is one of the common methods to enhance the therapeutic potential of MSCs and EVs they secrete [212]. Low oxygen levels in the environment enhance the MSC stemness and migration, with increased expression of HIF-1α playing a crucial role in boosting the therapeutic potential of the MSC secretome [213,214]. The studies on EV from MSC cultured under hypoxic conditions have shown enhanced proliferation and migration of chondrocytes due to the increased expression of miRNA-181c-5p, which suppresses the chondrocyte apoptosis through the miRNA-18-3P/JAK/STAT signaling pathway [212]. Despite the increased effectiveness of hypoxia in enhancing the therapeutic potential of MSC-derived EV, some researchers believe that priming MSC with growth factors is a more effective approach to modifying the therapeutic potential of MSC, which may also impact EV secreted by these cells [215].

### 4.2. Preconditioning with Pro-Inflammatory Factors and 3D Cultivation Conditions

One approach to modifying the content of MSC-derived EVs to increase their yield and composition involves modifying the cultivation conditions using the 3D culture environments. These methods create mechanical conditions for the cells that are closer to the natural conditions in terms of spatial and mechanical organization, maintaining the stemness of MSCs. One of the simplest approaches is the 3D cultivation of MSCs in the form of spheroids [211,216,217]. The use of spheroids as sources of EV significantly influences the therapeutic properties of EV, enhancing the immunosuppressive and regenerative effects of EV derived from cells cultured in the 3D conditions compared to the 2D conditions [211,218]. Exosomes from 3D cultures demonstrate higher regenerative potential in treating OA, enhancing chondrocyte migration and proliferation, and reducing cartilage tissue degeneration in animal models of OA [211].

MSC conditioning is a widely used strategy to modify both MSCs and EVs. It involves adding various pro-inflammatory cytokines, TLR agonists, and growth factors to MSCs [219,220]. Numerous studies have shown that IFN-γ, TNF-α, and IL-1β promote the formation of more therapeutically effective exosomes from MSCs for OA therapy [210,221]. Exosomes from ADMSC pre-treated with IFN-γ, TNF-α, and IL-1β influenced the secretion of exosomes carrying miR-24-3p, miR-222-3p, miR-34a-5p, and 146a-5p, which possess immunomodulatory properties affecting the macrophage polarization, promoting the formation of anti-inflammatory M2 macrophages, and positively influencing the deceleration of the ECM degradation processes [210,221]. EV from MSC preconditioned with lipopolysaccharide (LPS) more effectively suppressed the OA development, enhancing the chondrocyte proliferation and migration, and preventing the reduction in aggrecan and COL2A1 levels through the let-7b carried by EV [208]. Besides using pro-inflammatory molecules, MSC cultivation with growth factors and antioxidants is a strategy to enhance the regenerative potential of MSC-derived EV [222]. Priming BMSC with TGF-β1 led to exosomes with increased expression of miR-135b, which targeted MAPK6 to suppress the inflammatory process in mice with OA, enhancing the M2 macrophage polarization [223].

Summarizing the information from this section, altering the cultivation conditions of EV source cells could become a promising direction in EV modification. However, detailed studies are needed to understand the mechanisms enhancing the therapeutic potential of EV when the parent cells are exposed to various chemical and physical stimuli. The reliability and reproducibility of these effects also need further investigation. Additionally, more thorough research is required to compare EVs obtained through genetic modification of EV-producing cells with those obtained by modifying cultivation conditions.

### 4.3. Genetic and Drug Modifications

Genetic engineering is currently a common method to modify both MSCs and their secreted EVs to improve the therapeutic efficacy and targeting capabilities. Genetic engineering allows for the induction of stable expression of specific proteins, growth factors, cytokines, and various non-coding RNA molecules (miRNA, long non-coding RNA (lncRNA), circular RNA (circRNA)). Genetic engineering includes transfection using liposomes, electroporation, and other carriers, as well as viral vectors. Viral vectors are widely used to modify MSC and other cell cultures to obtain EVs with specific therapeutic properties for OA and RA therapy [224,225]. Viral vectors are characterized by a high transfection rate and high stability of gene synthesis, although the efficiency of viral transfection depends on the specific virus chosen and its ability to infect a particular target cell [226,227]. Lentiviral and adenoviral vectors are most commonly used, demonstrating a good safety profile and high transfection efficiency in MSC [228,229,230]. Lentiviral modification of BMSC increased the overexpression of lncRNA NEAT1, which alleviated OA manifestations in both in vitro and in vivo models [225]. NEAT1 binds to miR-122-5p, leading to the activation of the Sesn2/Nrf2 axis, positively affecting chondrocyte survival by enhancing proliferation and autophagy while inhibiting apoptosis [225]. Sesn2 levels decrease in tissues during OA development, reducing the ability to maintain chondrocyte survival, and its increased expression positively affects mTOR-dependent autophagy modulation [231].

In a study by Hong-Yan Meng et al., an adenoviral vector was used to obtain EV from MSC overexpressing miRNA-124a [232]. Modified exosomes with miRNA-124a targeted the suppression of FLS migration and proliferation by blocking the TNF-α-activated Ras-Erk1/2 pathway [232,233]. In addition to MSC, other cell sources are genetically engineered with viral vectors to obtain therapeutic exosomes carrying specific therapeutic molecules for RA therapy [224,234]. In a study by Paul D. Robbins et al., the immunosuppressive effect of exosomes derived from DC was enhanced by transducing DC with an adenovirus to overexpress IL-10 in the exosomes, which suppressed macroscopic changes in collagen-induced arthritis (CIA) in mice [224]. However, despite the effectiveness of viral modification methods, certain concerns remain because viruses can replicate in cells, potentially causing immune reactions, and the complexity and high costs of scaling this technology are significant limitations [235].

For the modification of MSC and other EV-producing cells for OA and RA therapy, non-viral methods such as the application of liposomes, polymeric carriers, electroporation, and ultrasound are often used [236,237]. Various small RNA molecules like miRNAs [238], circRNAs [239], and lncRNAs [240] are used as main cargos for EV modification. These molecules influence the biological functioning of recipient cells by interacting with target mRNAs or microRNAs, blocking the expression of genes related to immune regulation, inflammation, and enhanced cartilage tissue catabolism [241,242]. Excessive angiogenesis in RA promotes the synovial tissue’s hyperplasia and pannus formation, facilitating the migration of immune cells and amplifying inflammation, making it a key therapeutic target [243,244]. MiR-150-5p is significantly reduced in RA patients compared to OA patients. This microRNA is involved in angiogenesis regulation, and its deficiency influences the increased angiogenesis in RA patients [245]. Modification of BM-MSC exosomes by transfecting miR-150-5p suppresses the angiogenesis and hyperplasia of FLS in RA patients by reducing the expression of MMP-14 and vascular endothelial growth factor (VEGF) [246]. In RA, FLS can survive under high ROS conditions and exhibit abnormal proliferation under the oxidative stress caused by the induction of ferroptosis, characterized by iron-dependent lipid peroxidation [247]. The study by Zhiguo Lin et al. showed that EV from synovial MSC transfected with miR-433-3p could suppress angiogenesis by reducing the VEGF expression in human dermal microvascular endothelial cells, where angiogenesis was induced by EV from FLSs with elastin-induced ferroptosis [247]. Lipid nanoparticles were utilized to load synovial MSC for obtaining exosomes overexpressing miR-155-5p [248]. These exosomes stimulated chondrocyte migration and ECM secretion by targeting Runx2 via miR-155-5p, which increases synovial tissue in OA [248,249]. An alternative strategy for OA therapy involves stimulating the osteoblast survival, enhancing calcification, and increasing the secretion of osteogenesis markers such as osteocalcin and bone morphogenetic protein 2 by introducing exosomes from BM-MSC overexpressing miR-206 [250]. MiR-206 in exosomes targets the expression of the E74-like factor 3, a marker of inflammation and cartilage catabolic state in OA patients, and regulating its level may become one of the therapeutic strategies for OA therapy [250,251,252].

Transfection and transduction of miRNAs are widespread for genetic manipulation of cell recipient EV, but other types of nucleic acid cargos, such as circRNA [239,253] and lncRNA [240] are also used. The gene expression regulation mechanism of these molecules differs from miRNAs. circRNA represents a family of covalently closed ncRNA molecules and directly targets miRNA targets, allowing for a restricted inhibitory effect [254,255]. Exosomes from chondrogenically differentiated BM-MSC overexpressing circRNA_0001236 enhance anabolic processes in chondrocytes by targeting miR-3677-3p and Sox9, mitigating OA destructive processes in a DMM mouse model [256]. RA therapy with exosomes from MSC enriched with circFBXW7 can significantly suppress the inflammatory response of RA-FLS by absorbing miR-216a-3p, which releases histone deacetylase-4 activation, playing an inhibitory role in the RA inflammation progression [239]. Another circEDIL3 overexpressed in SMSC-EV inhibited the pathological angiogenesis in RA by reducing synovial VEGF expression induced by the IL-6/sIL-6R complex and suppressing the STAT3 activity, which plays a crucial role in the RA progression [257].

lncRNAs can regulate the development of OA and RA, acting similarly to circRNAs and inhibiting miRNAs that are overexpressed in these inflammatory arthritides, such as microRNA-29a-3p and miR-143-3p [258,259]. Besides reducing the destructive processes in the cartilage tissue in OA, an important direction in therapy is pain management. lncRNA H19 was enriched in exosomes from UCBMSC, and these EVs helped suppress pain syndrome and central sensitization in OA by targeting the lncRNA H19/miRNA-29a-3p/FOS axis [258]. Promising results were obtained in a study by Yuhua Su et al., where exosomes from MSC expressing HAND2-AS1 suppressed inflammation, proliferation, and induced cell death in RA-FLSs by inactivating the NF-κB pathway through the miR-143-3p/TNFAIP3 axis [259]. Despite the advances in genetic engineering of MSC to produce EV with predictable and specified therapeutic parameters, questions still remain regarding the stability of regulatory ncRNA expression and their ability to effectively reach recipient cells via therapeutic exosomes.

Stimulating the expression of regulatory ncRNAs is the most common strategy for modifying EV-producing cells and the exosomes themselves. Researchers also use other approaches, in which donor cells of EVs are stimulated to produce EVs with enhanced expression of immunoregulatory proteins. There are approaches where therapeutic molecules are loaded into EVs to increase their therapeutic efficacy and reduce unwanted side effects [204,260]. In a study by Seon Hee Kim et al., EVs from DC genetically modified to produce IL-4, which has both secreted and membrane-bound forms, were used [261]. Exosomes from IL-4-modified DC modulate the activity of antigen-presenting cells and T-cells in vivo through the MHC class II and partially via the Fas-ligand/Fas-dependent mechanism, positively affecting the progression of collagen-induced arthritis in vivo [261]. One approach to enhancing the synergistic chondrogenic effect of exosomes is their loading with kartogenin (KGN). This heterocyclic molecule is a promising candidate for stimulating chondrogenesis and anabolic processes in chondrocytes [262,263]. The addition of KGN to ADSC-derived exosomes has been shown to enhance the ADSC’s chondrogenic differentiation, reduce apoptosis, and suppress the expression of genes associated with the ECM degradation, including MMP-3, ADAMTS4, and ADAMTS5 [262]. Another approach to regulating inflammation in RA was targeting macrophage repolarization from M1 to M2. In this study, exosomes from M2 macrophages were additionally modified with a plasmid encoding IL-10 and betamethasone sodium phosphate [264]. This therapeutic system based on modified M2 macrophage- exosomes showed a powerful therapeutic effect in both in vitro and in vivo RA models, with EV having a good safety profile [264]. Table 1 provides more detailed information on the EV modification methods and their therapeutic effects in animal and cell models of OA and RA.

### 4.4. Surface Modification of EV

In recent years, various attempts have been made to develop therapeutic EVs as platforms for delivering their own therapeutic cargo and various drug molecules. However, there are several limitations, including a short half-life of EV up to 6 h with systemic administration and a tendency for EVs to accumulate in the liver and spleen, where they are metabolized [272,273,274]. The surface of EVs or EV-producing cells can be modified with various functional fragments by introducing surface proteins, peptides, enzymes, or chemical ligands that can covalently or non-covalently bind to proteins overexpressed in diseased or damaged tissues, increasing the targeting of EV to the desired therapeutic area [207,275,276]. One approach to cell surface modification is metabolic glycoengineering. In this method, cell metabolic pathways are modulated by introducing monosaccharide analogs into the cell’s metabolic pathways, resulting in the modification of the glycocalyx on the cell membrane surface, allowing for the creation of functional groups on the membrane surface [277,278]. This method has found wide application in immunotherapy and can also be applied to modify the surface of nanoparticles, enhancing their stability and targeting ability for EV cargo delivery [279,280,281]. Combining approaches in cell and EV surface modification with bioorthogonal chemistry and click chemistry with metabolic glycoengineering is effective. This method allows the addition of chemical groups to cell glycans [282]. Glycoengineering combined with bioorthogonal click chemistry has also been used to modify exosomes derived from ADSC for RA therapy. Surface modification of ADSC involved introducing an azide group via the metabolic glycoengineering pathway, followed by modifying the azide group with dibenzocyclooctyne dextran sulfate, enhancing exosome targeting to macrophages, and promoting their M2 polarization via the modulation of the JAK-STAT signaling pathway [205]. These modified exosomes had a ten times more pronounced therapeutic effect on the CIA treatment than unmodified ADSC exosomes did [205].

One important parameter in OA therapy using EVs is delivering the therapeutic cargo directly to chondrocytes. However, the dense ECM, significantly thicker in humans compared to small rodents often used as OA therapy models, poses a limitation for EVs reaching target cells [283]. Yujie Liang et al. developed modified EVs from DC, where they transfected a plasmid encoding a peptide’s affinity to chondrocytes associated with lysosomal membrane glycoprotein 2b. Additionally, these EVs were loaded with miR-140 via electroporation [284]. These genetically engineered EVs showed excellent results, demonstrating good distribution and therapeutic effect in the joint area, maintaining the presence for an extended period [284]. The therapeutic properties of UCMSC-derived EV were enhanced through dual engineering by increasing the expression of a peptide on the vesicle surface targeting collagen II, significantly improving the ability of these EVs to penetrate the dense cartilage ECM [238]. Isolated vesicles were further modified with miR-223 targeting NLRP3, the inhibition of which in OA and RA contributes to reduced secretion of IL-1β, TNF-α, and IL-18 by macrophages [238]. Various surface modification approaches are highly promising, potentially making EV therapy for OA and RA more effective, achieving therapeutic effects with significantly fewer EV injections into the joint area, a major advantage for patients with these pathologies.

### 4.5. Application of Tissue Bioengineering Approaches in EV Modification for Inflammatory Arthritis Therapy

EV therapy has shown promising results in cell and animal models of OA and RA. However, a key obstacle is the relatively low stability of EV upon systemic administration; for instance, intravenously administered exosomes accumulate in the liver and are rapidly cleared from the body [285,286]. A simple solution to this problem is injecting EV directly into the cartilage area. However, the efficacy and retention duration of vesicles in the cartilage area require detailed study. Some researchers are developing complex combined therapeutic systems that include not only exosomes but also nanocarriers or various scaffolds, which can enhance the targeting ability of exosomes to act on target cells and retain exosomes in the therapeutic area, ensuring uniform particle release, ultimately aiming to reduce the frequency of painful intra-articular injections [207,287]. In another study, hybrid nanoparticles mimicking exosomes were used as the therapeutic agent. These were obtained from M2 macrophages via extrusion, after which the membranes of these particles were fused with the membrane of M1 macrophages. This combination allowed the nanoparticles to retain the anti-inflammatory properties of M2 macrophages while the M1 macrophage membrane contained cytokine receptors for binding inflammatory factors [288]. To enhance the therapeutic effect, these nanovesicles were loaded with black phosphorus nanosheets, which, upon near-infrared irradiation, induced the death of inflammatory cells. This combined solution significantly suppressed inflammation while ensuring nanoparticle accumulation in the joints of mice with CIA [288]. To create a therapeutic system with enhanced accumulation and activation ability in the damaged organ area, systems sensitive to low pH, high enzyme activity (e.g., MMP), and ROS can be used [276]. In creating an EV carrier with enhanced targeting ability for RA therapy, researchers considered the elevated ROS levels in inflammatory joint diseases [207,289]. This system ensures a high potential for accumulation in the joint area upon intravenous administration to mice with CIA, due to the high ROS content. Tolerogenic DC-derived EV is released from a polyethylene glycol carrier in the joint area, creating an anti-inflammatory environment in the joint by reducing IL-6 levels and increasing CD4 + CD25 + Foxp3 + regulatory T-cells, providing a more pronounced immunoregulatory effect than non-modified EV [207].

Tissue engineering is a promising direction in therapy, combining biomaterials and exosomes to restore the cartilage structure, enhancing the therapeutic effect of EV through slow release and retention in the joint area [290]. Biomaterials of a natural origin, such as various hydrogels and scaffolds based on gelatin, chitosan, hyaluronic acid, peptides, and decellularized cartilage matrices, are the most widely used for the therapy of degenerative joint diseases [291,292]. Hydrogels provide a gradual exosome release during polymer swelling and subsequent degradation, allowing exosomes to easily diffuse through the dense extracellular matrix, a process facilitated by aquaporin-1 on the surface of EV, ensuring better distribution in the dense ECMs, such as the cartilage tissue, compared to synthetic nanoparticles [293,294]. Combining a gelatin methacryloyl scaffold with MSC-derived nanoparticles mimicking exosomes, obtained through MSC extrusion, demonstrated an outstanding retention time of EV in the joint area for more than seven days, promoting cartilage matrix restoration and creating an anti-inflammatory environment in vivo by enhancing macrophage polarization to the M2 phenotype [295]. A promising therapeutic strategy for treating degenerative cartilage diseases involves a combined system incorporating MSC-derived nanoparticles treated with KGN to amplify their therapeutic effect. When combined with 3D-bioprinted hydrogels, this system may enhance the synergistic effects of exosomes by providing immunomodulation and stimulating chondrogenesis via the exosomal cargo. Additionally, hydrogels can optimize the pharmacokinetics of EVs [296]. High therapeutic efficacy has been demonstrated for BMSC-derived exosomes treated with KGN, in combination with sodium alginate-based hydrogel and gelatin sponges, in modulating tendon regeneration. This approach enhanced the fibrochondral tissue regeneration [297,298]. The combination of exosomes with gelatin sponges allowed the in vivo retention of exosomes at the injury site for up to two weeks, while sodium alginate-based hydrogels extended retention to one week [297,298]. The integration of exosomes with kartogenin and an efficient exosome-release system promoted tendon healing and cartilage repair by enhancing the expression of glycosaminoglycans and collagen II, supporting enthesis regeneration. This combined approach has potential applications in cartilage repair for OA and RA [298].

**Figure 2:**
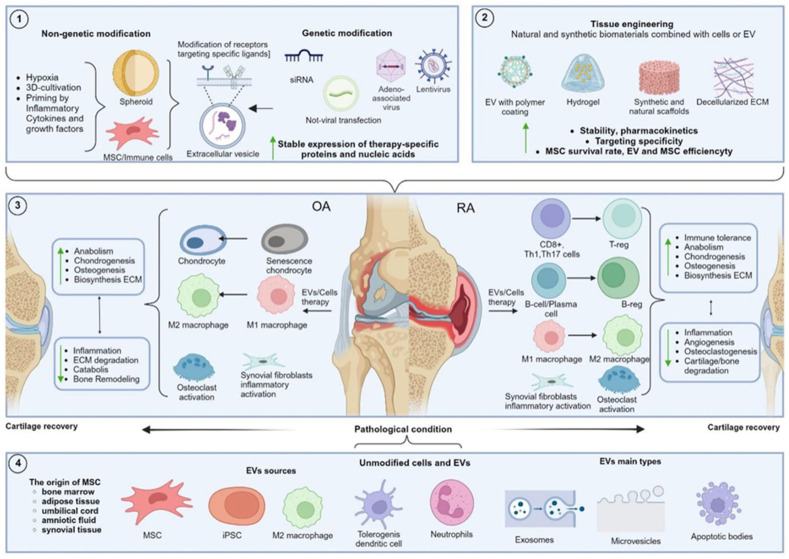
Summary of the approaches in OA and RA therapy discussed in this review, focusing on the use of EV and cell therapy.Blocks 1 and 2 describe the approaches to MSC and EV modification. Block 1 includes methods based on altering chemical and physical cultivation conditions, as well as key genetic engineering methods for modifying EV-producing cells. Block 2 outlines strategies for modifying cellular components or EV through the combination with various biomaterials. Block 3 shows the targets modified by EV and MSC in OA and RA treatment. Block 4 presents the main sources of unmodified EV and the classes of EV discussed in this review, depending on their biogenesis pathway.

Cationic modification of MSC-EV with the cationic amphiphilic macromolecule polyethylene distearoyl phosphatidylethanolamine allowed the construction of positively charged EV with efficient cartilage penetration, enhancing chondrocyte adsorption and achieving OA suppression with fewer injections compared to unmodified EV [266]. Another study created an innovative photoinduced imine cross-linked hydrogel adhesive, which retained up to 90% of exosomes within the gel for 14 days post-injection, enhancing the cartilage tissue regeneration and presenting a potential OA therapy that does not require frequent invasive joint interventions [299]. A promising approach involves creating a tissue-engineered construct incorporating MSC-derived exosomes and a decellularized cartilage matrix scaffold. This scaffold not only provides a favorable environment for endogenous chondrocyte migration but also delivers a rich cargo of miRNAs from MSC exosomes, suppressing cartilage inflammation and synergistically enhancing hyaline cartilage regeneration due to the favorable immune environment [291]. Tissue engineering approaches are highly promising alongside genetic and metabolic engineering of MSC sources to obtain modified exosomes. Combined approaches can ensure the success of clinical trials of these biological drugs, leading to the adoption of these technologies in clinical practice. The overview of approaches in OA and RA therapy using EV and cell therapy is presented in Figure 2.

## 5. Challenges and Prospects of Cell Therapy and MSC-Derived EV Therapy on the Path to Clinical Practice

In recent decades, a significant amount of data has been accumulated on the successful application of EV from various sources and MSC-based cell therapy, demonstrating therapeutic effects in cell and animal models of degenerative and inflammatory diseases such as OA and RA [175,184,300]. Unlike EV, MSC-based cell therapy for OA and RA is actively tested in clinical trials, with 52 trials for OA and 20 for RA (https://clinicaltrials.gov) [301]. Most clinical trials are in phase I/II, and at this stage, MSC injections have shown a good safety profile with a minimal incidence of side effects, mainly mild to moderate, such as arthralgia and joint swelling, which were resolved within a week [302,303,304]. In several studies, MSC administration in patients with RA and OA showed trends of clinical improvement, pain reduction, improved motor activity, and histological improvements in the cartilage tissue [305,306,307]. Thus, in a pilot study by Orozco et al., a single intra-articular injection of autologous bone marrow-derived MSC in knee OA patients led to a significant 65% improvement in WOMAC scores and MRI evidence of cartilage volume stabilization over 12 months [303]. Similarly, Garay-Mendoza et al. reported that autologous bone marrow MSC injections resulted in statistically significant improvements in both VAS pain scores and knee function scores, sustained over 6 months [305]. Bastos et al. conducted a double-blind clinical trial comparing MSC with and without PRP and found that both interventions significantly reduced knee pain and symptoms, though the addition of PRP did not confer a clear added benefit [307]. For RA, Shadmanfar et al. performed a randomized, triple-blind, placebo-controlled Phase I/II trial in patients with knee involvement and found that intra-articular MSC injections led to a marked decrease in DAS28 scores from 5.1 to 3.5 over 12 weeks, compared to a smaller reduction in the placebo group (5.0 to 4.5) [306]. Additionally, Álvaro-Gracia et al. demonstrated that intravenous administration of allogeneic adipose-derived MSC resulted in 65% of patients achieving ACR20 response at 12 weeks, versus 33% in the placebo group, along with a favorable safety profile [304]. Together, these trials consistently show that MSC therapy offers clinically meaningful benefits in both OA and RA patients, although the differences in the study design, MSC source, and treatment regimens underscore the need for standardized protocols and further comparative trials.

However, not all clinical trials demonstrate homogeneous and statistically significant clinical effects of MSC therapy for inflammatory joint diseases. Patients exhibited a positive trend in reducing clinical symptoms, but the effect was not statistically significant [308]. Regarding clinical trials of EV-based therapy for inflammatory diseases, this is still a relatively new field of research with a limited number of studies. Currently, there are no clinical trials using exosomes for RA [309]. Recent clinical investigations are exploring the potential of EVs as therapeutic agents for OA, highlighting a growing interest in their regenerative and immunomodulatory properties. Three Phase I trials are currently evaluating the safety and preliminary efficacy of EV in OA management (NCT06431152, NCT06463132, NCT04223622, NCT06937528). One study investigates dosage safety and joint function improvements (NCT06431152), while another examines safety, tolerability, and analgesic effects, focusing on pain reduction and enhanced mobility (NCT06463132). A third trial focuses on the safety and functional outcomes, particularly joint mobility and pain relief (NCT04223622). The clinical trial registered under ClinicalTrials.gov identifier NCT06937528 investigated the efficacy and safety of PMSC-EV in treating knee OA. All trials involve adults with knee OA, excluding those with systemic inflammatory conditions or recent joint interventions. The outcomes of these studies are anticipated to provide valuable insights into the therapeutic potential and clinical applicability of EVs for OA management.

MSC and MSC-derived EV therapy for OA and RA have shown successful results in animal and cell models of these diseases. However, clinical trials have yielded inconsistent results, often failing to achieve statistically significant therapeutic effects. Systematic reviews and meta-analyses have concluded that MSC therapy positively impacts the well-being of OA patients, but caution is warranted due to the low reliability of results [310,311]. Based on this result, several limitations and challenges on the path to clinical implementation of MSC and MSC-derived EV therapies need to be highlighted. Some problems lie at the level of clinical trial organization. Due to limited funding, several studies are conducted at single centers with small patient samples. Approximately half of the studies lacked a control group and did not employ patient “blinding.” Moreover, over 30% of the studies did not include patient randomization [312,313]. There are also concerns regarding preclinical studies, which predominantly use cell models and small laboratory animal models (mostly rats and mice). There are limited studies involving larger laboratory animals. This is crucial as the histological structure of large animal cartilage is closer to that of humans due to its greater thickness, affecting the parameters of vesicle and MSC biodistribution, requiring deeper ECM penetration, longer recovery periods, and different concentrations and frequencies of therapeutic agent administration [314]. Additionally, it is essential to understand that laboratory models of OA and RA may not reflect the full complexity of the diseases’ pathogenesis, leading to a lack of reproducibility of laboratory results in patients.

A common challenge for both MSC-based cell therapy and EV therapy derived from these cells is the problem of selecting the source of these cells. MSCs are highly heterogeneous among different donors, affecting their proliferation, differentiation, and therapeutic properties due to variations in gene expression, regulatory RNA cargo, and proteins secreted by MSC-EV [315,316]. An important parameter is the age of the donor, particularly for autologous MSCs, as they will differentiate into aging chondrocytes, which are less resilient to the pathological environment in OA and RA, since OA and, to a lesser extent, RA predominantly occur in the elderly [317,318]. The older age of the donor can also negatively affect MSC proliferation capacity, limiting these cells for EV production, as significant quantities of cells are required to achieve the therapeutic concentrations of EV, complicating large-scale EV production. MSCs have a limited number of passages, and with increased passages, they change their phenotype during prolonged passaging, which affects the composition of the exosomes they secrete [319].

The limited or absent proliferative capacity of immune cells used to obtain EV restricts their potential beyond the laboratory, despite their pronounced therapeutic effect in the RA and OA treatment [198,320]. Currently, there are no definitive standards for doses and frequency of EV and MSC administration to patients with degenerative joint diseases. An important direction is to determine the optimal doses for maximum efficacy and safety, as systemic administration of MSCs carries risks of thrombosis and embolization [321,322]. Exosomes and other EVs have fewer safety issues due to their lack of proliferative capacity and a lower risk of immune response [323,324]. However, their lower stability and smaller size affect their biodistribution and pharmacokinetics, necessitating careful evaluation of the dosage and modification of EV to enhance the stability and targeting efficiency of recipient cells. MSCs and EVs can be modified to improve their properties. Promising approaches combine genetic engineering and biomaterials with EV or MSC, enhancing the therapeutic cargo and pharmacokinetics. Standardizing protocols for cell cultivation, exosome isolation, and storage is essential. Clinical trials and large-scale production must consider the low stability of EVs. Optimal storage conditions are −80 °C for EV and −196 °C for cells, which incurs additional costs [325,326]. Low temperatures do not affect the therapeutic properties of EV, but MSCs require 24 h of cultivation post-cryopreservation to restore their therapeutic properties, complicating their preparation in a clinical setting [327].

The lack of research into the clinical application of extracellular vesicles is also associated with a number of challenges that need to be addressed to facilitate their introduction into medical practice [328]. A primary concern is the lack of standardized protocols for EV production, which complicates the assurance of product quality and consistency [329]. The International Society for Extracellular Vesicles has established a Regulatory Affairs Task Force to develop international standards and reporting guidelines for EVs, aiming to harmonize practices and ensure product reliability [330]. Assessing the safety and efficacy of EV-based therapies presents another significant challenge. Traditional preclinical and clinical evaluation methods may not fully capture the complexities of EV interactions within the human body. Innovative assessment techniques are necessary to accurately evaluate these therapies [331]. Regulatory agencies, such as the U.S. Food and Drug Administration (FDA), have recognized the need for clear guidelines concerning EV-based products [332]. For instance, the FDA has issued a public safety notification highlighting the lack of FDA-approved exosome products and warning against unapproved exosome therapies [332]. Additionally, the FDA has provided guidance on the regulatory considerations for human cells, tissues, and cellular and tissue-based products, which may be relevant to the development and approval of EV-based therapies [333].

Furthermore, the production and logistical complexities associated with EV-based therapies present significant challenges [334]. One of the major obstacles is the lack of standardized protocols for isolating and purifying EVs, which results in variability in their composition and functional properties [335]. The methods employed to isolate EV, such as ultracentrifugation, size-exclusion chromatography (SEC), filtration, and immunoaffinity-based approaches, each present distinct advantages and limitations that impact the consistency and quality of the vesicles [335,336]. Ultracentrifugation, the most commonly used method for EV isolation, is widely regarded as the “gold standard” due to its ability to generate high yields of EVs [337]. However, it requires specialized equipment and results in the co-isolation of contaminants, such as lipoproteins and protein aggregates, which can compromise the purity of the final EV preparation [338]. Additionally, the high g-forces involved in ultracentrifugation may lead to structural damage to the vesicles, thereby altering their biological activity and therapeutic potential [339]. SEC represents another commonly employed technique for EV isolation, particularly favored for its scalability and reproducibility. SEC separates vesicles based on their size and can be used to purify EVs from a variety of biological fluids [340]. This method is relatively gentle compared to ultracentrifugation, preserving the integrity of the EV. However, SEC typically results in lower yields than ultracentrifugation, and the separation process may not be sufficiently efficient for isolating vesicles from complex biological samples, leading to a less homogenous product [341]. The scalability of SEC is also limited by the need for specialized columns and the relatively high cost of materials, making it less practical for large-scale production [342]. Filtration is a simple and cost-effective technique for EV isolation, particularly for smaller volumes. Filters with defined pore sizes can capture EV while allowing smaller particles and contaminants to pass through [343]. While filtration is straightforward, it is not as efficient in terms of purity as more specialized techniques like SEC [335]. In addition, this method may not adequately capture the full spectrum of EVs, particularly those of varying sizes and densities, leading to less comprehensive isolation of vesicles [343]. Another significant challenge associated with filtration is the risk of membrane clogging, particularly when processing biological fluids with elevated concentrations of proteins and cellular debris [343]. Immunoaffinity-based isolation methods, which use antibodies targeting specific surface markers of EV, offer a highly targeted approach to isolating specific subpopulations of EV [344]. However, it is limited by the availability of suitable antibodies, and the technique can be time-consuming and expensive. Additionally, the reliance on specific markers may lead to the selective isolation of only certain subsets of EV, potentially missing important therapeutic components present in other populations of vesicles [344].

Large-scale production of MSC and EV preparations requires overcoming several challenges. For example, most research on MSC-derived EVs has been conducted using 2D models, which are unsuitable for large-scale production due to their low productivity, risk of contamination, and complexity in manufacturing organizations [345]. Promising approaches include using 3D cultures combined with scaffolds in hollow-fiber bioreactors, which can achieve a 100-fold increase in the exosome yield [211,346,347]. Hollow fiber bioreactor (HFB) systems, in particular, offer a promising platform for continuous, high-yield EV harvesting under controlled, GMP-compliant conditions. Gobin et al. demonstrated that MSC-derived EV produced in HFB systems retained a consistent particle size, immunophenotype, and functional characteristics over extended culture periods, supporting their clinical-grade reproducibility [348]. Similarly, Garcia et al. showed that immortalized MSCs maintained stable EV output and quality during prolonged bioreactor culture [349]. Moreover, the ability to modulate bioreactor environments, as shown by Ludlow et al., allows for selective tuning of the EV surface profiles, enhancing therapeutic customization [350]. The limited proliferative capacity of MSCs can be circumvented by replacing them with MSCs derived from induced pluripotent stem cells (iPSCs). iPSC-derived MSCs exhibit a younger phenotype and a high proliferative capacity of over 50 passages [351,352]. The use of iPSC-derived MSCs for exosome production is advantageous due to the inability of EVs to replicate; however, additional research is required to standardize the therapeutic cargo of exosomes. Combining these systems with iPSC-derived MSCs—which exhibit high proliferative potential and stable phenotypes—can offer a renewable, standardized source for EV production. An essential condition for industrial exosome production is the method of isolation. In laboratory settings, ultracentrifugation is commonly used, but it has a low yield and frequency of exosome recovery. Tangential flow filtration allows processing large volumes of the culture medium with a lower risk of losing the therapeutic properties of exosomes, making it advantageous for large-scale production [353].

## 6. Conclusions

The last two decades of research on MSCs and MSC-derived EVs have demonstrated high therapeutic potential for treating degenerative and inflammatory joint diseases. However, the complexity of cultivation and production, as well as the low stability of these sources for OA and RA therapy, has so far prevented MSCs and EVs from entering clinical practice. Despite many challenges, these therapeutic approaches are gradually progressing through clinical trials, with exosomes only taking their first steps. Despite all difficulties, there is confidence that EV-based preparations for OA and RA therapy will reach the market in the next 10–15 years. However, researchers still need to address several tasks, including creating optimized protocols for the cultivation, isolation, and storage of EVs, as well as selecting optimal therapeutic parameters at the patient level for RA and OA.

## Figures and Tables

**Table 1:** Approaches to EV modification and their therapeutic effect on in vitro and in vivo models of OA and RA.

Type Arthritis	Model of Arthritis/Type of Animal	Source EV	Cargo	The Cells Modification/EV Approach	Method of Administration, Concentration	Therapeutic Effects		Mechanism	Ref.
						↑	↓		
OA	DMM and ACLT	BMSC	hsa-miR-181c-5p, hsa-miR-18a-3p	Hypoxic conditions	Intraarticular injections, 100 µg protein per 100 µL PBS	Chondrocyte proliferation and migration	Apoptosis in IL-1β-treated chondrocytes OARSI scores	miRNA-18-3P/JAK/STAT or miRNA-181c-5p/MAPK pathways	[212]
OA	MLI	BMSC	miR-135b	Stimulation with TGF-β1	Intraarticular injections, 1 × 10^11^ exosome particles/mL 100 µL PBS	EV-BMSC: Arg-1 and iNOS in macrophages	EV-BMSC-TGF-β1: IL-1β, PGE2, COX-2, COX-1, and NO in serum	EV-BMSC-TGF-β1: overexpression of miR-135b, M2 macrophage polarization via the MAPK6 pathway	[223]
RA	CIA, mice	BMSC	miR-205-5p	Chondrogenic differentiation	Tail vein injections, 200 µL PBS	-	TNF-α, IL-6, MMP1, and MMP13 in IL-1β-treated FLSs. MDM2, IL-1β, IL-6, and TNF-α in the serum	miR-205-5 expression, MAPK, and NF-κB pathways inhibition through MDM2	[184]
OA	CIOA	BMSC	miR-92a-3p	Chondrogenic differentiation	Intraarticular injections, 15 µL of EV in PBS (500 µg/mL).	COL2A1, aggrecan	MMP13	miR-92a-3p by targeting WNT5A	[265]
OA	DMM	BMSC	NEAT1	Transduction with a lentivirus for NEAT1 overexpression	Intraarticular injections, 10 µg protein per dose	Chondrocyte proliferation and autophagy	Apoptosis	Binding miR-122-5p, activating the Sesn2/Nrf2 axis	[225]
OA	Intraarticular injections of cold water (4 °C)	SMSC	miR-155-5p	Transfection of miR-155-5p using Lipofectamine 2000 (Invitrogen; Thermo Fisher Scientific, Inc., Carlsbad, CA, USA).	Intraarticular injections, 30 μL; 10^11^ exo particles/mL PBS	Proliferation, migration, and ECM secretion in chondrocytes; number of chondrocytes	OARSI scores	By targeting Runx2	[248]
OA	ACLT	BMSC	miR-206	Transfection of miR-206 using Lipofectamine 2000 (Invitrogen; Thermo Fisher Scientific, Inc., Carlsbad, CA, USA)	Intraarticular injections, 10^11^ EV/mL, 100 µL PBS	OCN and BMP2, alkaline phosphatase activity, calcium deposition	Apoptosis in OA osteoblasts	miR-206-overexpression by downregulating the E74-like factor 3	[250]
OA	MIA	UCMSC	miR-223	(1) Transduction with a lentivirus encoding CTP-Lamp2b (2) exosome loading with miR-223 by electroporation	Intraarticular injections, 10^10^ particles/mL, 50 µL PBS	Col II and Sox9 in chondrocytes	MMP13, NLRP3, IL-1β, MMP2, IL-1β, TNF-α, and PGE2	via the miR-223/NLRP3/pyroptosis axis	[238]
OA	DMM	BMSC	circRNA_0001236	Transfection of a plasmid encoding circRNA_0001236	Intraarticular injections, 500 µg/mL EV circRNA_0001236, 10 µL PBS	Col2a1 and Sox9 in human chondrocyte cartilage tissues	MMP13	circRNA_0001236 through the miR-3677-3p/Sox9 axis	[256]
OA	ACLT	(iPSC) derived MSC	-	Surface charge by polyethylene distearoyl phosphatidylethanolamine incorporation	Intraarticular injections, modified and unmodified EV at a particle concentration of (1 × 10^9^ particles/mL; 1 × 10^10^ particles/mL)	Chondrocyte uptake, Col2, aggrecan	IL-1β-induced apoptosis MMP13 and ADAMTS5	-	[266]
RA	CIA	BMSC	FGL-1	Transfection with an FGL1 plasmid using Lipofectamine 2000 (Invitrogen; Thermo Fisher Scientific, Inc., Carlsbad, CA, USA)	-	-	Apoptosis in FLSs. MMP-9, IL-8, IL-1β, and IL-17 levels in serum	By inhibiting the NF-κB transduction	[185]
OA	DMM	IPFP	miR-100-5p	-	Intraarticular injections, 10 µL MSC-IPFP-EV (10^10^ particles/mL)	Col2	ADAMTS5 and MMP13 IL-1β-induced apoptosis in chondrocytes	miR-100-5p inhibits the mTOR-autophagy pathway	[267]
OA	ACLT	BMSC	miR-361-5p	miR-NC and miR-361-5p by electroporation	Intraarticular injections, Exo-miR-NC and Exo-miR-361-5p concentration of 250 ng/5 µL of PBS	-	iNOS, MMP-3, MMP-13, IL-18, IL-6, and TNF-α	miR-361-5p inhibits NF-κB signaling by suppressing DDX20	[268]
RA	CIA	DC	Indolamine-2,3-dioxygenase IDO	Transduction with adenovirus expressing IDO	Intravenous injections, DC-Exo/DC-Exo-IDO concentration 1 μg protein in 20 μL of PBS	-	The arthritis index	The costimulatory molecules B7	[234]
RA	CIA	BMMSC	miR-34a	Transfection with miR-34a inhibitor using Lipofectamine 2000 (Invitrogen; Thermo Fisher Scientific, Inc., Carlsbad, CA, USA)	Intravenous injections, BMMSC-EV 75 µg/mL of PBS	RA-FLS apoptosis	TNF-α, IL-6, and IL-8 mRNA in synovial tissue and synovial fluid. RA-FLS proliferation and increased	By inhibiting the Cyclin I/ATM/ATR/p53 signaling pathway	[269]
RA	CIA	GMDSC	miR-29a-3p and miR-93-5p	Transfection with miR-29a-3p and miR-93-5p with Entranster-R (Engreen Biosystem Co., Ltd., Beijing, China)	Intravenous injections, GMDSC-EV 100 μg/mouse	-	the mean arthritis index IFN-γ and IL-17A in the serum Th1 and Th17 differentiation in vitro	-	[197]
RA	CIA	BMMSC	miR-320a	Transfection with miR-320a plasmids using Lipofectamine 2000 (Invitrogen; Thermo Fisher Scientific, Inc., Carlsbad, CA, USA)	Intravenous injections, BMMSC-EV 100 µg/day	-	IL-1β, IL-6, and IL-8 in RA-FLSs	Overexpressed miR-320a by suppressing CXCL9 expression	[270]
OA	ACLT/MCLT	BMMSC	miR-9-5p	Transfection with mimic and inhibitors miR-9-5p using Lipofectamine 2000 (Invitrogen; Thermo Fisher Scientific, Inc., Carlsbad, CA, USA)	Intraarticular injections, BMMSC -EV, or liposomes containing miR-9-5p mimic/inhibitor	-	IL-1, IL-6, TNF-α, and CRP oxidative stress indicators (NO, iNOS, COX2, and SOD) MMP-13 and OCN in synovial fluid	Regulation of the expression of syndecan-1	[271]
OA	ACLT	M2-Mφ	miR-21-5p	-	Intraarticular injections, M1-AB and M2-AB 10 µg/10 µL	M2-AB: IL-4 and IL-10 in M1 macrophages M1-ABs: CD-86 in synovial tissue, cartilage thickness	M2-Ab: IL-1β, IL-6, IL-17, TNF-α, IFN-γ, and MCP-1 CD206 in synovial tissue	-	[201]

Abbreviations used in the table. ↑—increase; ↓—decrease: AB—apoptotic bodies; ACLT—Anterior Cruciate Ligament Transection; BMP2—bone morphogenetic protein 2; BMSC—bone mesenchymal stem cells; CIA—collagen-induced arthritis; CIOA—collagenase-induced osteoarthritis; FGL1—fibrinogen-like protein 1; IPFP-MSC—infrapatellar fat pad MSC; Mφ—macrophage; MCLT—medial and collateral ligament transection; MIA—Monosodium Iodoacetate; OARSI—Osteoarthritis Research Society International.

## Data Availability

Data generated during the study and included in this article are available from the corresponding authors upon request.

## Abbreviations

ACLT—Anterior Cruciate Ligament Transection; AMP—adenosine monophosphate; ADAMTS-5—ADAM metallopeptidase with thrombospondin type 1 motif 5; ATMSC—Adipose tissue mesenchymal stem cells; AB—apoptotic bodies; BMSC—bone mesenchymal stem cells; BMMSC—bone marrow mesenchymal stem cells; BMP—bone morphogenetic protein; CD—cluster of differentiation; CIA—collagen-induced arthritis; Col—collagen; CXCL-9—C-X-C motif chemokine ligand9; DMARD—disease-modifying antirheumatic drug; ECM—extracellular matrix; ERK—signal-regulated kinases; EV—extracellular vesicles; FDA—U.S. Food and Drug Administration; FGL1—fibrinogen-like protein 1; FLS—fibroblast-like synoviocytes; GMSC—gingival mesenchymal stem cells; GMDSC—granulocytic myeloid-derived suppressor cells; HFB—hollow fiber bioreactor; HIF-1α—hypoxia-inducible factor-1α; HLA—human leukocyte antigen; ICAM-1—inter-cellular adhesion molecule 1; IDO—indoleamine-pyrrole 2,3-dioxygenase; iPSC—induced pluripotent stem cells; IPFP-MSC—Infrapatellar fat pad; IL—interleukin; KGN—kartogenin; LPS—lipopolysaccharide; M-CSF—macrophage colony-stimulating factor; Mφ—macrophage; MBV—matrix-bound nanovesicles; MCP-1—monocyte chemoattractant protein 1; MCLT—medial and collateral ligament transection; METTL3—N6-adenosine-methyltransferase 70 kDa subunit; MIA—monosodium iodoacetate; MSC—mesenchymal stem cells; MHC—major histocompatibility complex; miRNA—MicroRNA; mRNA—messenger RNA; mTOR—mammalian target of rapamycin; NLRP3—nod-like-receptor family pyrin domain containing 3; NF-κB—Nuclear factor kappa-light-chain-enhancer of activated B cells; NOX4—NADPH oxidase 4; NSAID—nonsteroidal anti-inflammatory drug; NRF2—nuclear factor erythroid 2-related factor 2; OA—osteoarthritis; PD–1—programmed cell death protein 1; RA—rheumatoid arthritis; RANKL—receptor activator of nuclear factor kappa-Β ligand; ROS—reactive oxygen species; SESN2—Sestrin-2; SEC—size-exclusion chromatography; Sox9—sex-determining region of the Y-chromosome—transcription factor 9; STAT—signal transducer and activator of transcription; tDC—tolerogenic dendritic cells; TGF-β—transforming growth factor beta; Th—T helper cells; TLR—Toll-like receptor; TNF-α—tumor necrosis factor-α; Treg—regulatory T cells; UCMSC—umbilical cord mesenchymal stem cells; VEGF—vascular endothelial growth factor.

## References

[1] Lin Y.-J., Anzaghe M., Schülke S. (2020). Update on the Pathomechanism, Diagnosis, and Treatment Options for Rheumatoid Arthritis. Cells.

[2] Hunter D.J., March L., Chew M. (2020). Osteoarthritis in 2020 and beyond: A Lancet Commission. Lancet.

[3] Aletaha D., Smolen J.S. (2018). Diagnosis and Management of Rheumatoid Arthritis: A Review. JAMA.

[4] Myasoedova E., Davis J., Matteson E.L., Crowson C.S. (2020). Is the Epidemiology of Rheumatoid Arthritis Changing? Results from a Population-Based Incidence Study, 1985–2014. Ann. Rheum. Dis..

[5] Zhao X., Shah D., Gandhi K., Wei W., Dwibedi N., Webster L., Sambamoorthi U. (2019). Clinical, Humanistic, and Economic Burden of Osteoarthritis among Noninstitutionalized Adults in the United States. Osteoarthr. Cartil..

[6] Katz J.N., Arant K.R., Loeser R.F. (2021). Diagnosis and Treatment of Hip and Knee Osteoarthritis: A Review. JAMA.

[7] Conforti A., Di Cola I., Pavlych V., Ruscitti P., Berardicurti O., Ursini F., Giacomelli R., Cipriani P. (2021). Beyond the Joints, the Extra-Articular Manifestations in Rheumatoid Arthritis. Autoimmun. Rev..

[8] Raychaudhuri S., Sandor C., Stahl E.A., Freudenberg J., Lee H.-S., Jia X., Alfredsson L., Padyukov L., Klareskog L., Worthington J. (2012). Five Amino Acids in Three HLA Proteins Explain Most of the Association between MHC and Seropositive Rheumatoid Arthritis. Nat. Genet..

[9] Darrah E., Andrade F. (2018). Rheumatoid Arthritis and Citrullination. Curr. Opin. Rheumatol..

[10] Sen R., Hurley J.A. (2024). Osteoarthritis. StatPearls.

[11] Gelber A.C., Hochberg M.C., Mead L.A., Wang N.-Y., Wigley F.M., Klag M.J. (2000). Joint Injury in Young Adults and Risk for Subsequent Knee and Hip Osteoarthritis. Ann. Intern. Med..

[12] Sahin V., Karakaş E.S., Aksu S., Atlihan D., Turk C.Y., Halici M. (2003). Traumatic Dislocation and Fracture-Dislocation of the Hip: A Long-Term Follow-up Study. J. Trauma.

[13] Abramoff B., Caldera F.E. (2020). Osteoarthritis: Pathology, Diagnosis, and Treatment Options. Med. Clin. N. Am..

[14] Mort J.S., Billington C.J. (2001). Articular Cartilage and Changes in Arthritis: Matrix Degradation. Arthritis Res. Ther..

[15] Xia B., Chen D., Zhang J., Hu S., Jin H., Tong P. (2014). Osteoarthritis Pathogenesis: A Review of Molecular Mechanisms. Calcif. Tissue Int..

[16] Lambert C., Zappia J., Sanchez C., Florin A., Dubuc J.-E., Henrotin Y. (2021). The Damage-Associated Molecular Patterns (DAMPs) as Potential Targets to Treat Osteoarthritis: Perspectives from a Review of the Literature. Front. Med..

[17] Neogi T. (2012). Clinical Significance of Bone Changes in Osteoarthritis. Ther. Adv. Musculoskelet. Dis..

[18] Buckley C.D., McGettrick H.M. (2018). Leukocyte Trafficking between Stromal Compartments: Lessons from Rheumatoid Arthritis. Nat. Rev. Rheumatol..

[19] Alivernini S., MacDonald L., Elmesmari A., Finlay S., Tolusso B., Gigante M.R., Petricca L., Di Mario C., Bui L., Perniola S. (2020). Distinct Synovial Tissue Macrophage Subsets Regulate Inflammation and Remission in Rheumatoid Arthritis. Nat. Med..

[20] Zhao S., Grieshaber-Bouyer R., Rao D.A., Kolb P., Chen H., Andreeva I., Tretter T., Lorenz H.-M., Watzl C., Wabnitz G. (2022). Effect of JAK Inhibition on the Induction of Proinflammatory HLA–DR+CD90+ Rheumatoid Arthritis Synovial Fibroblasts by Interferon-γ. Arthritis Rheumatol..

[21] Floudas A., Neto N., Orr C., Canavan M., Gallagher P., Hurson C., Monaghan M.G., Nagpar S., Mullan R.H., Veale D.J. (2022). Loss of Balance between Protective and Pro-Inflammatory Synovial Tissue T-Cell Polyfunctionality Predates Clinical Onset of Rheumatoid Arthritis. Ann. Rheum. Dis..

[22] Smolen J.S., Aletaha D., Barton A., Burmester G.R., Emery P., Firestein G.S., Kavanaugh A., McInnes I.B., Solomon D.H., Strand V. (2018). Rheumatoid Arthritis. Nat. Rev. Dis. Primer.

[23] Luo G., Li F., Li X., Wang Z.-G., Zhang B. (2018). TNF-α and RANKL Promote Osteoclastogenesis by Upregulating RANK via the NF-κB Pathway. Mol. Med. Rep..

[24] Meednu N., Zhang H., Owen T., Sun W., Wang V., Cistrone C., Rangel-Moreno J., Xing L., Anolik J.H. (2016). Production of RANKL by Memory B Cells. Arthritis Rheumatol..

[25] Ouboussad L., Burska A.N., Melville A., Buch M.H. (2019). Synovial Tissue Heterogeneity in Rheumatoid Arthritis and Changes with Biologic and Targeted Synthetic Therapies to Inform Stratified Therapy. Front. Med..

[26] Peng X., Chen X., Zhang Y., Tian Z., Wang M., Chen Z. (2025). Advances in the Pathology and Treatment of Osteoarthritis. J. Adv. Res..

[27] Han P., Liu X., He J., Han L., Li J. (2024). Overview of Mechanisms and Novel Therapies on Rheumatoid Arthritis from a Cellular Perspective. Front. Immunol..

[28] Coaccioli S., Sarzi-Puttini P., Zis P., Rinonapoli G., Varrassi G. (2022). Osteoarthritis: New Insight on Its Pathophysiology. J. Clin. Med..

[29] Bannuru R.R., Osani M.C., Vaysbrot E.E., Arden N.K., Bennell K., Bierma-Zeinstra S.M.A., Kraus V.B., Lohmander L.S., Abbott J.H., Bhandari M. (2019). OARSI Guidelines for the Non-Surgical Management of Knee, Hip, and Polyarticular Osteoarthritis. Osteoarthr. Cartil..

[30] Archer R., Hock E., Hamilton J., Stevens J., Essat M., Poku E., Clowes M., Pandor A., Stevenson M. (2018). Assessing Prognosis and Prediction of Treatment Response in Early Rheumatoid Arthritis: Systematic Reviews. Health Technol. Assess. Winch. Engl..

[31] Kerola A.M., Rollefstad S., Semb A.G. (2021). Atherosclerotic Cardiovascular Disease in Rheumatoid Arthritis: Impact of Inflammation and Antirheumatic Treatment. Eur. Cardiol. Rev..

[32] Gomez E.A., Colas R.A., Souza P.R., Hands R., Lewis M.J., Bessant C., Pitzalis C., Dalli J. (2020). Blood Pro-Resolving Mediators Are Linked with Synovial Pathology and Are Predictive of DMARD Responsiveness in Rheumatoid Arthritis. Nat. Commun..

[33] Bindu S., Mazumder S., Bandyopadhyay U. (2020). Non-Steroidal Anti-Inflammatory Drugs (NSAIDs) and Organ Damage: A Current Perspective. Biochem. Pharmacol..

[34] Kloppenburg M., Berenbaum F. (2020). Osteoarthritis Year in Review 2019: Epidemiology and Therapy. Osteoarthr. Cartil..

[35] Tschopp M., Pfirrmann C.W.A., Fucentese S.F., Brunner F., Catanzaro S., Kühne N., Zwyssig I., Sutter R., Götschi T., Tanadini M. (2023). A Randomized Trial of Intra-Articular Injection Therapy for Knee Osteoarthritis. Investig. Radiol..

[36] Robinson P.D., McEwan J., Adukia V., Prabhakar M. (2018). Osteoarthritis and Arthroplasty of the Hip and Knee. Br. J. Hosp. Med..

[37] Regmi S., Pathak S., Kim J.O., Yong C.S., Jeong J.-H. (2019). Mesenchymal Stem Cell Therapy for the Treatment of Inflammatory Diseases: Challenges, Opportunities, and Future Perspectives. Eur. J. Cell Biol..

[38] Chung M.-J., Son J.-Y., Park S., Park S.-S., Hur K., Lee S.-H., Lee E.-J., Park J.-K., Hong I.-H., Kim T.-H. (2021). Mesenchymal Stem Cell and MicroRNA Therapy of Musculoskeletal Diseases. Int. J. Stem Cells.

[39] Ragni E., Perucca Orfei C., De Luca P., Mondadori C., Viganò M., Colombini A., de Girolamo L. (2020). Inflammatory Priming Enhances Mesenchymal Stromal Cell Secretome Potential as a Clinical Product for Regenerative Medicine Approaches through Secreted Factors and EV-miRNAs: The Example of Joint Disease. Stem Cell Res. Ther..

[40] Hernigou P., Bouthors C., Bastard C., Flouzat Lachaniette C.H., Rouard H., Dubory A. (2021). Subchondral Bone or Intra-Articular Injection of Bone Marrow Concentrate Mesenchymal Stem Cells in Bilateral Knee Osteoarthritis: What Better Postpone Knee Arthroplasty at Fifteen Years? A Randomized Study. Int. Orthop..

[41] Liu H., Li R., Liu T., Yang L., Yin G., Xie Q. (2020). Immunomodulatory Effects of Mesenchymal Stem Cells and Mesenchymal Stem Cell-Derived Extracellular Vesicles in Rheumatoid Arthritis. Front. Immunol..

[42] Dominici M., Le Blanc K., Mueller I., Slaper-Cortenbach I., Marini F., Krause D., Deans R., Keating A., Prockop D., Horwitz E. (2006). Minimal Criteria for Defining Multipotent Mesenchymal Stromal Cells. The International Society for Cellular Therapy Position Statement. Cytotherapy.

[43] Kobolak J., Dinnyes A., Memic A., Khademhosseini A., Mobasheri A. (2016). Mesenchymal Stem Cells: Identification, Phenotypic Characterization, Biological Properties and Potential for Regenerative Medicine through Biomaterial Micro-Engineering of Their Niche. Methods.

[44] Ilas D.C., Churchman S.M., Baboolal T., Giannoudis P.V., Aderinto J., McGonagle D., Jones E. (2019). The Simultaneous Analysis of Mesenchymal Stem Cells and Early Osteocytes Accumulation in Osteoarthritic Femoral Head Sclerotic Bone. Rheumatology.

[45] Turajane T., Chaveewanakorn U., Fongsarun W., Aojanepong J., Papadopoulos K.I. (2017). Avoidance of Total Knee Arthroplasty in Early Osteoarthritis of the Knee with Intra-Articular Implantation of Autologous Activated Peripheral Blood Stem Cells versus Hyaluronic Acid: A Randomized Controlled Trial with Differential Effects of Growth Factor Addition. Stem Cells Int..

[46] Wu X., Wang W., Meng C., Yang S., Duan D., Xu W., Liu X., Tang M., Wang H. (2013). Regulation of Differentiation in Trabecular Bone-derived Mesenchymal Stem Cells by T Cell Activation and Inflammation. Oncol. Rep..

[47] Neybecker P., Henrionnet C., Pape E., Mainard D., Galois L., Loeuille D., Gillet P., Pinzano A. (2018). In Vitro and in Vivo Potentialities for Cartilage Repair from Human Advanced Knee Osteoarthritis Synovial Fluid-Derived Mesenchymal Stem Cells. Stem Cell Res. Ther..

[48] Greif D.N., Kouroupis D., Murdock C.J., Griswold A.J., Kaplan L.D., Best T.M., Correa D. (2020). Infrapatellar Fat Pad/Synovium Complex in Early-Stage Knee Osteoarthritis: Potential New Target and Source of Therapeutic Mesenchymal Stem/Stromal Cells. Front. Bioeng. Biotechnol..

[49] Chen X., Zheng J., Yin L., Li Y., Liu H. (2024). Transplantation of Three Mesenchymal Stem Cells for Knee Osteoarthritis, Which Cell and Type Are More Beneficial? A Systematic Review and Network Meta-Analysis. J. Orthop. Surg..

[50] Lee W.-S., Kim H.J., Kim K.-I., Kim G.B., Jin W. (2019). Intra-Articular Injection of Autologous Adipose Tissue-Derived Mesenchymal Stem Cells for the Treatment of Knee Osteoarthritis: A Phase IIb, Randomized, Placebo-Controlled Clinical Trial. Stem Cells Transl. Med..

[51] Freitag J., Bates D., Wickham J., Shah K., Huguenin L., Tenen A., Paterson K., Boyd R. (2019). Adipose-Derived Mesenchymal Stem Cell Therapy in the Treatment of Knee Osteoarthritis: A Randomized Controlled Trial. Regen. Med..

[52] Wang Z.-G., He Z.-Y., Liang S., Yang Q., Cheng P., Chen A.-M. (2020). Comprehensive Proteomic Analysis of Exosomes Derived from Human Bone Marrow, Adipose Tissue, and Umbilical Cord Mesenchymal Stem Cells. Stem Cell Res. Ther..

[53] Wagenbrenner M., Heinz T., Horas K., Jakuscheit A., Arnholdt J., Herrmann M., Rudert M., Holzapfel B.M., Steinert A.F., Weißenberger M. (2020). The Human Arthritic Hip Joint Is a Source of Mesenchymal Stromal Cells (MSCs) with Extensive Multipotent Differentiation Potential. BMC Musculoskelet. Disord..

[54] Yu Y., Yoon K.-A., Kang T.-W., Jeon H.-J., Sim Y.-B., Choe S.H., Baek S.Y., Lee S., Seo K.-W., Kang K.-S. (2019). Therapeutic Effect of Long-Interval Repeated Intravenous Administration of Human Umbilical Cord Blood-Derived Mesenchymal Stem Cells in DBA/1 Mice with Collagen-Induced Arthritis. J. Tissue Eng. Regen. Med..

[55] Gowhari Shabgah A., Shariati-Sarabi Z., Tavakkol-Afshari J., Ghasemi A., Ghoryani M., Mohammadi M. (2020). A Significant Decrease of BAFF, APRIL, and BAFF Receptors Following Mesenchymal Stem Cell Transplantation in Patients with Refractory Rheumatoid Arthritis. Gene.

[56] Sobacchi C., Palagano E., Villa A., Menale C. (2017). Soluble Factors on Stage to Direct Mesenchymal Stem Cells Fate. Front. Bioeng. Biotechnol..

[57] Su J., Chen X., Huang Y., Li W., Li J., Cao K., Cao G., Zhang L., Li F., Roberts A.I. (2014). Phylogenetic Distinction of iNOS and IDO Function in Mesenchymal Stem Cell-Mediated Immunosuppression in Mammalian Species. Cell Death Differ..

[58] Sun A.R., Panchal S.K., Friis T., Sekar S., Crawford R., Brown L., Xiao Y., Prasadam I. (2017). Obesity-Associated Metabolic Syndrome Spontaneously Induces Infiltration of pro-Inflammatory Macrophage in Synovium and Promotes Osteoarthritis. PLoS ONE.

[59] Weyand C.M., Goronzy J.J. (2021). The Immunology of Rheumatoid Arthritis. Nat. Immunol..

[60] Liu B., Zhang M., Zhao J., Zheng M., Yang H. (2018). Imbalance of M1/M2 Macrophages Is Linked to Severity Level of Knee Osteoarthritis. Exp. Ther. Med..

[61] Liu K.-J., Wang C.-J., Chang C.-J., Hu H.-I., Hsu P.-J., Wu Y.-C., Bai C.-H., Sytwu H.-K., Yen B.L. (2011). Surface Expression of HLA-G Is Involved in Mediating Immunomodulatory Effects of Placenta-Derived Multipotent Cells (PDMCs) towards Natural Killer Lymphocytes. Cell Transplant..

[62] Selmani Z., Naji A., Zidi I., Favier B., Gaiffe E., Obert L., Borg C., Saas P., Tiberghien P., Rouas-Freiss N. (2008). Human Leukocyte Antigen-G5 Secretion by Human Mesenchymal Stem Cells Is Required to Suppress T Lymphocyte and Natural Killer Function and to Induce CD4+CD25highFOXP3+ Regulatory T Cells. Stem Cells.

[63] Schurgers E., Kelchtermans H., Mitera T., Geboes L., Matthys P. (2010). Discrepancy between the in Vitro and in Vivo Effects of Murine Mesenchymal Stem Cells on T-Cell Proliferation and Collagen-Induced Arthritis. Arthritis Res. Ther..

[64] Fan X.-L., Zhang Z., Ma C.Y., Fu Q.-L. (2019). Mesenchymal Stem Cells for Inflammatory Airway Disorders: Promises and Challenges. Biosci. Rep..

[65] Song N., Scholtemeijer M., Shah K. (2020). Mesenchymal Stem Cell Immunomodulation: Mechanisms and Therapeutic Potential. Trends Pharmacol. Sci..

[66] Hong J., Hueckelhoven A., Wang L., Schmitt A., Wuchter P., Tabarkiewicz J., Kleist C., Bieback K., Ho A.D., Schmitt M. (2016). Indoleamine 2,3-Dioxygenase Mediates Inhibition of Virus-Specific CD8^+^ T Cell Proliferation by Human Mesenchymal Stromal Cells. Cytotherapy.

[67] Hwu P., Du M.X., Lapointe R., Do M., Taylor M.W., Young H.A. (2000). Indoleamine 2,3-Dioxygenase Production by Human Dendritic Cells Results in the Inhibition of T Cell Proliferation. J. Immunol..

[68] Duffy M.M., Pindjakova J., Hanley S.A., McCarthy C., Weidhofer G.A., Sweeney E.M., English K., Shaw G., Murphy J.M., Barry F.P. (2011). Mesenchymal Stem Cell Inhibition of T-helper 17 Cell- Differentiation Is Triggered by Cell–Cell Contact and Mediated by Prostaglandin E2 via the EP4 Receptor. Eur. J. Immunol..

[69] Steffen U., Schett G., Bozec A. (2019). How Autoantibodies Regulate Osteoclast Induced Bone Loss in Rheumatoid Arthritis. Front. Immunol..

[70] Arai F., Miyamoto T., Ohneda O., Inada T., Sudo T., Brasel K., Miyata T., Anderson D.M., Suda T. (1999). Commitment and Differentiation of Osteoclast Precursor Cells by the Sequential Expression of C-Fms and Receptor Activator of Nuclear Factor kappaB (RANK) Receptors. J. Exp. Med..

[71] Xing L., Xiu Y., Boyce B.F. (2012). Osteoclast Fusion and Regulation by RANKL-Dependent and Independent Factors. World J. Orthop..

[72] Hamdalla H.M., Ahmed R.R., Galaly S.R., Ahmed O.M., Naguib I.A., Alghamdi B.S., Abdul-Hamid M. (2022). Assessment of the Efficacy of Bone Marrow-Derived Mesenchymal Stem Cells against a Monoiodoacetate-Induced Osteoarthritis Model in Wistar Rats. Stem Cells Int..

[73] Shin T.-H., Kim H.-S., Kang T.-W., Lee B.-C., Lee H.-Y., Kim Y.-J., Shin J.-H., Seo Y., Won Choi S., Lee S. (2016). Human Umbilical Cord Blood-Stem Cells Direct Macrophage Polarization and Block Inflammasome Activation to Alleviate Rheumatoid Arthritis. Cell Death Dis..

[74] Schelbergen R.F., van Dalen S., ter Huurne M., Roth J., Vogl T., Noël D., Jorgensen C., van den Berg W.B., van de Loo F.A., Blom A.B. (2014). Treatment Efficacy of Adipose-Derived Stem Cells in Experimental Osteoarthritis Is Driven by High Synovial Activation and Reflected by S100A8/A9 Serum Levels. Osteoarthr. Cartil..

[75] Choi H., Lee R.H., Bazhanov N., Oh J.Y., Prockop D.J. (2011). Anti-Inflammatory Protein TSG-6 Secreted by Activated MSCs Attenuates Zymosan-Induced Mouse Peritonitis by Decreasing TLR2/NF-κB Signaling in Resident Macrophages. Blood.

[76] Melief S.M., Geutskens S.B., Fibbe W.E., Roelofs H. (2013). Multipotent Stromal Cells Skew Monocytes towards an Anti-Inflammatory Interleukin-10-Producing Phenotype by Production of Interleukin-6. Haematologica.

[77] Song W.-J., Li Q., Ryu M.-O., Ahn J.-O., Ha Bhang D., Chan Jung Y., Youn H.-Y. (2017). TSG-6 Secreted by Human Adipose Tissue-Derived Mesenchymal Stem Cells Ameliorates DSS-Induced Colitis by Inducing M2 Macrophage Polarization in Mice. Sci. Rep..

[78] François M., Romieu-Mourez R., Li M., Galipeau J. (2012). Human MSC Suppression Correlates with Cytokine Induction of Indoleamine 2,3-Dioxygenase and Bystander M2 Macrophage Differentiation. Mol. Ther..

[79] Oshita K., Yamaoka K., Udagawa N., Fukuyo S., Sonomoto K., Maeshima K., Kurihara R., Nakano K., Saito K., Okada Y. (2011). Human Mesenchymal Stem Cells Inhibit Osteoclastogenesis through Osteoprotegerin Production. Arthritis Rheum..

[80] Galgaro B.C., Beckenkamp L.R., Nunnenkamp M.v.d.M., Korb V.G., Naasani L.I.S., Roszek K., Wink M.R. (2021). The Adenosinergic Pathway in Mesenchymal Stem Cell Fate and Functions. Med. Res. Rev..

[81] Saldanha-Araujo F., Ferreira F.I.S., Palma P.V., Araujo A.G., Queiroz R.H.C., Covas D.T., Zago M.A., Panepucci R.A. (2011). Mesenchymal Stromal Cells Up-Regulate CD39 and Increase Adenosine Production to Suppress Activated T-Lymphocytes. Stem Cell Res..

[82] Pinto-Cardoso R., Pereira-Costa F., Pedro Faria J., Bandarrinha P., Bessa-Andrês C., Correia-de-Sá P., Bernardo Noronha-Matos J. (2020). Adenosinergic Signalling in Chondrogenesis and Cartilage Homeostasis: Friend or Foe?. Biochem. Pharmacol..

[83] Strazzulla L.C., Cronstein B.N. (2016). Regulation of Bone and Cartilage by Adenosine Signaling. Purinergic Signal..

[84] Hazrati A., Malekpour K., Khorramdelazad H., Rajaei S., Hashemi S.M. (2024). Therapeutic and Immunomodulatory Potentials of Mesenchymal Stromal/Stem Cells and Immune Checkpoints Related Molecules. Biomark. Res..

[85] Corciulo C., Lendhey M., Wilder T., Schoen H., Cornelissen A.S., Chang G., Kennedy O.D., Cronstein B.N. (2017). Endogenous Adenosine Maintains Cartilage Homeostasis and Exogenous Adenosine Inhibits Osteoarthritis Progression. Nat. Commun..

[86] Luo Y., Wu W., Gu J., Zhang X., Dang J., Wang J., Zheng Y., Huang F., Yuan J., Xue Y. (2019). Human Gingival Tissue-Derived MSC Suppress Osteoclastogenesis and Bone Erosion via CD39-Adenosine Signal Pathway in Autoimmune Arthritis. eBioMedicine.

[87] Ilchovska D., Barrow D.M. (2021). An Overview of the NF-kB Mechanism of Pathophysiology in Rheumatoid Arthritis, Investigation of the NF-kB Ligand RANKL and Related Nutritional Interventions. Autoimmun. Rev..

[88] Zimmermann J.A., Hettiaratchi M.H., McDevitt T.C. (2017). Enhanced Immunosuppression of T Cells by Sustained Presentation of Bioactive Interferon-γ Within Three-Dimensional Mesenchymal Stem Cell Constructs. Stem Cells Transl. Med..

[89] Fan H., Zhao G., Liu L., Liu F., Gong W., Liu X., Yang L., Wang J., Hou Y. (2012). Pre-Treatment with IL-1β Enhances the Efficacy of MSC Transplantation in DSS-Induced Colitis. Cell. Mol. Immunol..

[90] Bétous R., Renoud M.-L., Hoede C., Gonzalez I., Jones N., Longy M., Sensebé L., Cazaux C., Hoffmann J.-S. (2017). Human Adipose-Derived Stem Cells Expanded Under Ambient Oxygen Concentration Accumulate Oxidative DNA Lesions and Experience Procarcinogenic DNA Replication Stress. Stem Cells Transl. Med..

[91] Estrada J.C., Albo C., Benguría A., Dopazo A., López-Romero P., Carrera-Quintanar L., Roche E., Clemente E.P., Enríquez J.A., Bernad A. (2012). Culture of Human Mesenchymal Stem Cells at Low Oxygen Tension Improves Growth and Genetic Stability by Activating Glycolysis. Cell Death Differ..

[92] Lee S.-G., Joe Y.A. (2018). Autophagy Mediates Enhancement of Proangiogenic Activity by Hypoxia in Mesenchymal Stromal/Stem Cells. Biochem. Biophys. Res. Commun..

[93] Wang Q., Yang Q., Wang Z., Tong H., Ma L., Zhang Y., Shan F., Meng Y., Yuan Z. (2016). Comparative Analysis of Human Mesenchymal Stem Cells from Fetal-Bone Marrow, Adipose Tissue, and Warton’s Jelly as Sources of Cell Immunomodulatory Therapy. Hum. Vaccines Immunother..

[94] Ruiz M., Toupet K., Maumus M., Rozier P., Jorgensen C., Noël D. (2020). TGFBI Secreted by Mesenchymal Stromal Cells Ameliorates Osteoarthritis and Is Detected in Extracellular Vesicles. Biomaterials.

[95] Uzieliene I., Bernotas P., Mobasheri A., Bernotiene E. (2018). The Role of Physical Stimuli on Calcium Channels in Chondrogenic Differentiation of Mesenchymal Stem Cells. Int. J. Mol. Sci..

[96] Parate D., Franco-Obregón A., Fröhlich J., Beyer C., Abbas A.A., Kamarul T., Hui J.H.P., Yang Z. (2017). Enhancement of Mesenchymal Stem Cell Chondrogenesis with Short-Term Low Intensity Pulsed Electromagnetic Fields. Sci. Rep..

[97] Steward A.J., Kelly D.J. (2015). Mechanical Regulation of Mesenchymal Stem Cell Differentiation. J. Anat..

[98] Mueller A.-L., Payandeh Z., Mohammadkhani N., Mubarak S.M.H., Zakeri A., Alagheband Bahrami A., Brockmueller A., Shakibaei M. (2021). Recent Advances in Understanding the Pathogenesis of Rheumatoid Arthritis: New Treatment Strategies. Cells.

[99] Lee D.H., Kim S.J., Kim S.A., Ju G.-I. (2022). Past, Present, and Future of Cartilage Restoration: From Localized Defect to Arthritis. Knee Surg. Relat. Res..

[100] Deng Y., Lu J., Li W., Wu A., Zhang X., Tong W., Ho K.K., Qin L., Song H., Mak K.K. (2018). Reciprocal Inhibition of YAP/TAZ and NF-κB Regulates Osteoarthritic Cartilage Degradation. Nat. Commun..

[101] Mehana E.-S.E., Khafaga A.F., El-Blehi S.S. (2019). The Role of Matrix Metalloproteinases in Osteoarthritis Pathogenesis: An Updated Review. Life Sci..

[102] Chen Y., Cheng R.-J., Wu Y., Huang D., Li Y., Liu Y. (2023). Advances in Stem Cell-Based Therapies in the Treatment of Osteoarthritis. Int. J. Mol. Sci..

[103] Zhang Q., Xiang E., Rao W., Zhang Y.Q., Xiao C.H., Li C.Y., Han B., Wu D. (2021). Intra-Articular Injection of Human Umbilical Cord Mesenchymal Stem Cells Ameliorates Monosodium Iodoacetate-Induced Osteoarthritis in Rats by Inhibiting Cartilage Degradation and Inflammation. Bone Jt. Res..

[104] Wang A.-T., Zhang Q.-F., Wang N.-X., Yu C.-Y., Liu R.-M., Luo Y., Zhao Y.-J., Xiao J.-H. (2020). Cocktail of Hyaluronic Acid and Human Amniotic Mesenchymal Cells Effectively Repairs Cartilage Injuries in Sodium Iodoacetate-Induced Osteoarthritis Rats. Front. Bioeng. Biotechnol..

[105] Guan M., Yu Q., Zhou G., Wang Y., Yu J., Yang W., Li Z. (2024). Mechanisms of Chondrocyte Cell Death in Osteoarthritis: Implications for Disease Progression and Treatment. J. Orthop. Surg..

[106] Dickinson S.C., Sutton C.A., Brady K., Salerno A., Katopodi T., Williams R.L., West C.C., Evseenko D., Wu L., Pang S. (2017). The Wnt5a Receptor, Receptor Tyrosine Kinase-Like Orphan Receptor 2, Is a Predictive Cell Surface Marker of Human Mesenchymal Stem Cells with an Enhanced Capacity for Chondrogenic Differentiation. Stem Cells.

[107] Wang H., Yan X., Jiang Y., Wang Z., Li Y., Shao Q. (2018). The Human Umbilical Cord Stem Cells Improve the Viability of OA Degenerated Chondrocytes. Mol. Med. Rep..

[108] Jeon H.-J., Yoon K.-A., An E.S., Kang T.-W., Sim Y.-B., Ahn J., Choi E.-K., Lee S., Seo K.-W., Kim Y.-B. (2020). Therapeutic Effects of Human Umbilical Cord Blood-Derived Mesenchymal Stem Cells Combined with Cartilage Acellular Matrix Mediated via Bone Morphogenic Protein 6 in a Rabbit Model of Articular Cruciate Ligament Transection. Stem Cell Rev. Rep..

[109] Lin S., Lee W.Y.W., Xu L., Wang Y., Chen Y., Ho K.K.W., Qin L., Jiang X., Cui L., Li G. (2017). Stepwise Preconditioning Enhances Mesenchymal Stem Cell-Based Cartilage Regeneration through Epigenetic Modification. Osteoarthr. Cartil..

[110] Bolduc J.A., Collins J.A., Loeser R.F. (2019). Reactive Oxygen Species, Aging and Articular Cartilage Homeostasis. Free Radic. Biol. Med..

[111] Rousset F., Hazane-Puch F., Pinosa C., Nguyen M.V.C., Grange L., Soldini A., Rubens-Duval B., Dupuy C., Morel F., Lardy B. (2015). IL-1beta Mediates MMP Secretion and IL-1beta Neosynthesis via Upregulation of P22phox and NOX4 Activity in Human Articular Chondrocytes. Osteoarthr. Cartil..

[112] Platas J., Guillén M.I., del Caz M.D.P., Gomar F., Castejón M.A., Mirabet V., Alcaraz M.J. (2016). Paracrine Effects of Human Adipose-Derived Mesenchymal Stem Cells in Inflammatory Stress-Induced Senescence Features of Osteoarthritic Chondrocytes. Aging.

[113] Coryell P.R., Diekman B.O., Loeser R.F. (2021). Mechanisms and Therapeutic Implications of Cellular Senescence in Osteoarthritis. Nat. Rev. Rheumatol..

[114] Wang R., Maimaitijuma T., Ma Y.-Y., Jiao Y., Cao Y.-P. (2021). Mitochondrial Transfer from Bone-Marrow-Derived Mesenchymal Stromal Cells to Chondrocytes Protects against Cartilage Degenerative Mitochondrial Dysfunction in Rats Chondrocytes. Chin. Med. J..

[115] Souza A.G., Silva I.B.B., Campos-Fernandez E., Barcelos L.S., Souza J.B., Marangoni K., Goulart L.R., Alonso-Goulart V. (2018). Comparative Assay of 2D and 3D Cell Culture Models: Proliferation, Gene Expression and Anticancer Drug Response. Curr. Pharm. Des..

[116] Bartosh T.J., Ylöstalo J.H., Mohammadipoor A., Bazhanov N., Coble K., Claypool K., Lee R.H., Choi H., Prockop D.J. (2010). Aggregation of Human Mesenchymal Stromal Cells (MSCs) into 3D Spheroids Enhances Their Antiinflammatory Properties. Proc. Natl. Acad. Sci. USA.

[117] Wong C.-C., Sheu S.-D., Chung P.-C., Yeh Y.-Y., Chen C.-H., Chang Y.-W., Kuo T.-F. (2021). Hyaluronic Acid Supplement as a Chondrogenic Adjuvant in Promoting the Therapeutic Efficacy of Stem Cell Therapy in Cartilage Healing. Pharmaceutics.

[118] Joyce K., Fabra G.T., Bozkurt Y., Pandit A. (2021). Bioactive Potential of Natural Biomaterials: Identification, Retention and Assessment of Biological Properties. Signal Transduct. Target. Ther..

[119] Zhan X. (2019). Effect of Matrix Stiffness and Adhesion Ligand Density on Chondrogenic Differentiation of Mesenchymal Stem Cells. J. Biomed. Mater. Res. Part A.

[120] Shao J., Ding Z., Li L., Chen Y., Zhu J., Qian Q. (2020). Improved Accumulation of TGF-β by Photopolymerized Chitosan/Silk Protein Bio-Hydrogel Matrix to Improve Differentiations of Mesenchymal Stem Cells in Articular Cartilage Tissue Regeneration. J. Photochem. Photobiol. B.

[121] Baldari S., Di Rocco G., Piccoli M., Pozzobon M., Muraca M., Toietta G. (2017). Challenges and Strategies for Improving the Regenerative Effects of Mesenchymal Stromal Cell-Based Therapies. Int. J. Mol. Sci..

[122] Park Y.-B., Ha C.-W., Lee C.-H., Yoon Y.C., Park Y.-G. (2017). Cartilage Regeneration in Osteoarthritic Patients by a Composite of Allogeneic Umbilical Cord Blood-Derived Mesenchymal Stem Cells and Hyaluronate Hydrogel: Results from a Clinical Trial for Safety and Proof-of-Concept with 7 Years of Extended Follow-Up. Stem Cells Transl. Med..

[123] Gonzalez-Fernandez P., Simula L., Jenni S., Jordan O., Allémann E. (2024). Hyaluronan-Based Hydrogel Delivering Glucose to Mesenchymal Stem Cells Intended to Treat Osteoarthritis. Int. J. Pharm..

[124] Volarevic V., Markovic B.S., Gazdic M., Volarevic A., Jovicic N., Arsenijevic N., Armstrong L., Djonov V., Lako M., Stojkovic M. (2018). Ethical and Safety Issues of Stem Cell-Based Therapy. Int. J. Med. Sci..

[125] Ljujic B., Milovanovic M., Volarevic V., Murray B., Bugarski D., Przyborski S., Arsenijevic N., Lukic M.L., Stojkovic M. (2013). Human Mesenchymal Stem Cells Creating an Immunosuppressive Environment and Promote Breast Cancer in Mice. Sci. Rep..

[126] Gazdic M., Volarevic V., Arsenijevic N., Stojkovic M. (2015). Mesenchymal Stem Cells: A Friend or Foe in Immune-Mediated Diseases. Stem Cell Rev. Rep..

[127] Zhu X., Ma D., Yang B., An Q., Zhao J., Gao X., Zhang L. (2023). Research Progress of Engineered Mesenchymal Stem Cells and Their Derived Exosomes and Their Application in Autoimmune/Inflammatory Diseases. Stem Cell Res. Ther..

[128] Musiał-Wysocka A., Kot M., Majka M. (2019). The Pros and Cons of Mesenchymal Stem Cell-Based Therapies. Cell Transplant..

[129] Deng Y., Zhang Y., Ye L., Zhang T., Cheng J., Chen G., Zhang Q., Yang Y. (2016). Umbilical Cord-Derived Mesenchymal Stem Cells Instruct Monocytes Towards an IL10-Producing Phenotype by Secreting IL6 and HGF. Sci. Rep..

[130] Davies L.C., Heldring N., Kadri N., Le Blanc K. (2017). Mesenchymal Stromal Cell Secretion of Programmed Death-1 Ligands Regulates T Cell Mediated Immunosuppression. Stem Cells.

[131] Lai J.J., Chau Z.L., Chen S.-Y., Hill J.J., Korpany K.V., Liang N.-W., Lin L.-H., Lin Y.-H., Liu J.K., Liu Y.-C. (2022). Exosome Processing and Characterization Approaches for Research and Technology Development. Adv. Sci..

[132] Smirnova O., Efremov Y., Klyucherev T., Peshkova M., Senkovenko A., Svistunov A., Timashev P. (2024). Direct and Cell-Mediated EV-ECM Interplay. Acta Biomater..

[133] Jeppesen D.K., Zhang Q., Franklin J.L., Coffey R.J. (2023). Extracellular Vesicles and Nanoparticles: Emerging Complexities. Trends Cell Biol..

[134] Kalluri R., LeBleu V.S. (2020). The Biology, Function, and Biomedical Applications of Exosomes. Science.

[135] Welsh J.A., Goberdhan D.C.I., O’Driscoll L., Buzas E.I., Blenkiron C., Bussolati B., Cai H., Di Vizio D., Driedonks T.A.P., Erdbrügger U. (2024). Minimal Information for Studies of Extracellular Vesicles (MISEV2023): From Basic to Advanced Approaches. J. Extracell. Vesicles.

[136] Willms E., Cabañas C., Mäger I., Wood M.J.A., Vader P. (2018). Extracellular Vesicle Heterogeneity: Subpopulations, Isolation Techniques, and Diverse Functions in Cancer Progression. Front. Immunol..

[137] Hessvik N.P., Llorente A. (2018). Current Knowledge on Exosome Biogenesis and Release. Cell. Mol. Life Sci. CMLS.

[138] Zhao R., Zhao T., He Z., Cai R., Pang W. (2021). Composition, Isolation, Identification and Function of Adipose Tissue-Derived Exosomes. Adipocyte.

[139] Yáñez-Mó M., Siljander P.R.-M., Andreu Z., Zavec A.B., Borràs F.E., Buzas E.I., Buzas K., Casal E., Cappello F., Carvalho J. (2015). Biological Properties of Extracellular Vesicles and Their Physiological Functions. J. Extracell. Vesicles.

[140] Caruso S., Poon I.K.H. (2018). Apoptotic Cell-Derived Extracellular Vesicles: More Than Just Debris. Front. Immunol..

[141] Dostert G., Mesure B., Menu P., Velot É. (2017). How Do Mesenchymal Stem Cells Influence or Are Influenced by Microenvironment through Extracellular Vesicles Communication?. Front. Cell Dev. Biol..

[142] Wang L., Wang C., Jia X., Yu J. (2018). Circulating Exosomal miR-17 Inhibits the Induction of Regulatory T Cells via Suppressing TGFBR II Expression in Rheumatoid Arthritis. Cell. Physiol. Biochem..

[143] Yan J., Shen M., Sui B., Lu W., Han X., Wan Q., Liu Y., Kang J., Qin W., Zhang Z. (2022). Autophagic LC3+ Calcified Extracellular Vesicles Initiate Cartilage Calcification in Osteoarthritis. Sci. Adv..

[144] Zeng G., Deng G., Xiao S., Li F. (2022). Fibroblast-like Synoviocytes-Derived Exosomal PCGEM1 Accelerates IL-1β-Induced Apoptosis and Cartilage Matrix Degradation by miR-142-5p/RUNX2 in Chondrocytes. Immunol. Investig..

[145] Kolhe R., Hunter M., Liu S., Jadeja R.N., Pundkar C., Mondal A.K., Mendhe B., Drewry M., Rojiani M.V., Liu Y. (2017). Gender-Specific Differential Expression of Exosomal miRNA in Synovial Fluid of Patients with Osteoarthritis. Sci. Rep..

[146] Michael B.N.R., Kommoju V., Kavadichanda Ganapathy C., Negi V.S. (2019). Characterization of Cell-Derived Microparticles in Synovial Fluid and Plasma of Patients with Rheumatoid Arthritis. Rheumatol. Int..

[147] Jouybari M.T., Mojtahedi F., Babaahmadi M., Faeed M., Eslaminejad M.B., Taghiyar L. (2024). Advancements in Extracellular Vesicle Targeted Therapies for Rheumatoid Arthritis: Insights into Cellular Origins, Current Perspectives, and Emerging Challenges. Stem Cell Res. Ther..

[148] Jeon O.H., Wilson D.R., Clement C.C., Rathod S., Cherry C., Powell B., Lee Z., Khalil A.M., Green J.J., Campisi J. (2019). Senescence Cell–Associated Extracellular Vesicles Serve as Osteoarthritis Disease and Therapeutic Markers. JCI Insight.

[149] Wu X., Crawford R., Xiao Y., Mao X., Prasadam I. (2021). Osteoarthritic Subchondral Bone Release Exosomes That Promote Cartilage Degeneration. Cells.

[150] Arntz O.J., Pieters B.C.H., Thurlings R.M., Wenink M.H., van Lent P.L.E.M., Koenders M.I., van den Hoogen F.H.J., van der Kraan P.M., van de Loo F.A.J. (2018). Rheumatoid Arthritis Patients with Circulating Extracellular Vesicles Positive for IgM Rheumatoid Factor Have Higher Disease Activity. Front. Immunol..

[151] Burbano C., Villar-Vesga J., Vásquez G., Muñoz-Vahos C., Rojas M., Castaño D. (2019). Proinflammatory Differentiation of Macrophages Through Microparticles That Form Immune Complexes Leads to T- and B-Cell Activation in Systemic Autoimmune Diseases. Front. Immunol..

[152] Song J.E., Kim J.S., Shin J.H., Moon K.W., Park J.K., Park K.S., Lee E.Y. (2021). Role of Synovial Exosomes in Osteoclast Differentiation in Inflammatory Arthritis. Cells.

[153] Neumann E., Lefèvre S., Zimmermann B., Gay S., Müller-Ladner U. (2010). Rheumatoid Arthritis Progression Mediated by Activated Synovial Fibroblasts. Trends Mol. Med..

[154] Frank-Bertoncelj M., Pisetsky D.S., Kolling C., Michel B.A., Gay R.E., Jüngel A., Gay S. (2018). TLR3 Ligand Poly(I:C) Exerts Distinct Actions in Synovial Fibroblasts When Delivered by Extracellular Vesicles. Front. Immunol..

[155] Cavallo C., Merli G., Borzì R.M., Zini N., D’Adamo S., Guescini M., Grigolo B., Di Martino A., Santi S., Filardo G. (2021). Small Extracellular Vesicles from Adipose Derived Stromal Cells Significantly Attenuate in Vitro the NF-κB Dependent Inflammatory/Catabolic Environment of Osteoarthritis. Sci. Rep..

[156] Lu L., Wang J., Fan A., Wang P., Chen R., Lu L., Yin F. (2021). Synovial Mesenchymal Stem Cell-Derived Extracellular Vesicles Containing microRN555A-26a-5p Ameliorate Cartilage Damage of Osteoarthritis. J. Gene Med..

[157] Tian X., Wei W., Cao Y., Ao T., Huang F., Javed R., Wang X., Fan J., Zhang Y., Liu Y. (2022). Gingival Mesenchymal Stem Cell-Derived Exosomes Are Immunosuppressive in Preventing Collagen-Induced Arthritis. J. Cell. Mol. Med..

[158] Wu X., Zhu D., Tian J., Tang X., Guo H., Ma J., Xu H., Wang S. (2020). Granulocytic Myeloid-Derived Suppressor Cell Exosomal Prostaglandin E2 Ameliorates Collagen-Induced Arthritis by Enhancing IL-10+ B Cells. Front. Immunol..

[159] Cosenza S., Ruiz M., Toupet K., Jorgensen C., Noël D. (2017). Mesenchymal Stem Cells Derived Exosomes and Microparticles Protect Cartilage and Bone from Degradation in Osteoarthritis. Sci. Rep..

[160] Varderidou-Minasian S., Lorenowicz M.J. (2020). Mesenchymal Stromal/Stem Cell-Derived Extracellular Vesicles in Tissue Repair: Challenges and Opportunities. Theranostics.

[161] Kim G.B., Shon O.-J., Seo M.-S., Choi Y., Park W.T., Lee G.W. (2021). Mesenchymal Stem Cell-Derived Exosomes and Their Therapeutic Potential for Osteoarthritis. Biology.

[162] Casado-Díaz A., Quesada-Gómez J.M., Dorado G. (2020). Extracellular Vesicles Derived From Mesenchymal Stem Cells (MSC) in Regenerative Medicine: Applications in Skin Wound Healing. Front. Bioeng. Biotechnol..

[163] Elahi F.M., Farwell D.G., Nolta J.A., Anderson J.D. (2020). Preclinical Translation of Exosomes Derived from Mesenchymal Stem/Stromal Cells. Stem Cells.

[164] Sun L., Xu R., Sun X., Duan Y., Han Y., Zhao Y., Qian H., Zhu W., Xu W. (2016). Safety Evaluation of Exosomes Derived from Human Umbilical Cord Mesenchymal Stromal Cell. Cytotherapy.

[165] Chang T.-H., Wu C.-S., Chiou S.-H., Chang C.-H., Liao H.-J. (2022). Adipose-Derived Stem Cell Exosomes as a Novel Anti-Inflammatory Agent and the Current Therapeutic Targets for Rheumatoid Arthritis. Biomedicines.

[166] Alvarez-Erviti L., Seow Y., Yin H., Betts C., Lakhal S., Wood M.J.A. (2011). Delivery of siRNA to the Mouse Brain by Systemic Injection of Targeted Exosomes. Nat. Biotechnol..

[167] Zhang J., Li S., Li L., Li M., Guo C., Yao J., Mi S. (2015). Exosome and Exosomal microRNA: Trafficking, Sorting, and Function. Genom. Proteom. Bioinform..

[168] Vader P., Mol E.A., Pasterkamp G., Schiffelers R.M. (2016). Extracellular Vesicles for Drug Delivery. Adv. Drug Deliv. Rev..

[169] Lee E.S., Ko H., Kim C.H., Kim H.-C., Choi S.-K., Jeong S.W., Lee S.-G., Lee S.-J., Na H.-K., Park J.H. (2023). Disease-Microenvironment Modulation by Bare- or Engineered-Exosome for Rheumatoid Arthritis Treatment. Biomater. Res..

[170] Mellado M., Martínez-Muñoz L., Cascio G., Lucas P., Pablos J.L., Rodríguez-Frade J.M. (2015). T Cell Migration in Rheumatoid Arthritis. Front. Immunol..

[171] Lotfi N., Thome R., Rezaei N., Zhang G.-X., Rezaei A., Rostami A., Esmaeil N. (2019). Roles of GM-CSF in the Pathogenesis of Autoimmune Diseases: An Update. Front. Immunol..

[172] Wang B., Zhao P., Zhou Y., Meng L., Zhu W., Jiang C., Wang L., Cai Y., Lu S., Hou W. (2017). Increased Expression of Th17 Cytokines and Interleukin-22 Correlates with Disease Activity in Pristane-Induced Arthritis in Rats. PLoS ONE.

[173] Chang L., Feng X., Gao W. (2019). Proliferation of Rheumatoid Arthritis Fibroblast-like Synoviocytes Is Enhanced by IL-17-Mediated Autophagy through STAT3 Activation. Connect. Tissue Res..

[174] Liu C., Qian W., Qian Y., Giltiay N.V., Lu Y., Swaidani S., Misra S., Deng L., Chen Z.J., Li X. (2009). Act1, a U-Box E3 Ubiquitin Ligase for IL-17 Signaling. Sci. Signal..

[175] Ma D., Xu K., Zhang G., Liu Y., Gao J., Tian M., Wei C., Li J., Zhang L. (2019). Immunomodulatory Effect of Human Umbilical Cord Mesenchymal Stem Cells on T Lymphocytes in Rheumatoid Arthritis. Int. Immunopharmacol..

[176] Cosenza S., Toupet K., Maumus M., Luz-Crawford P., Blanc-Brude O., Jorgensen C., Noël D. (2018). Mesenchymal Stem Cells-Derived Exosomes Are More Immunosuppressive than Microparticles in Inflammatory Arthritis. Theranostics.

[177] Komatsu N., Takayanagi H. (2022). Mechanisms of Joint Destruction in Rheumatoid Arthritis—Immune Cell-Fibroblast-Bone Interactions. Nat. Rev. Rheumatol..

[178] Koedderitzsch K., Zezina E., Li L., Herrmann M., Biesemann N. (2021). TNF Induces Glycolytic Shift in Fibroblast like Synoviocytes via GLUT1 and HIF1A. Sci. Rep..

[179] Zhang B., Gu J., Wang Y., Guo L., Xie J., Yang M. (2023). TNF-α Stimulated Exosome Derived from Fibroblast-like Synoviocytes Isolated from Rheumatoid Arthritis Patients Promotes HUVEC Migration, Invasion and Angiogenesis by Targeting the miR-200a-3p/KLF6/VEGFA Axis. Autoimmunity.

[180] Yoshitomi H. (2019). Regulation of Immune Responses and Chronic Inflammation by Fibroblast-Like Synoviocytes. Front. Immunol..

[181] del Rey M.J., Izquierdo E., Caja S., Usategui A., Santiago B., Galindo M., Pablos J.L. (2009). Human Inflammatory Synovial Fibroblasts Induce Enhanced Myeloid Cell Recruitment and Angiogenesis through a Hypoxia-Inducible Transcription Factor 1alpha/Vascular Endothelial Growth Factor-Mediated Pathway in Immunodeficient Mice. Arthritis Rheum..

[182] Machado C.R.L., Dias F.F., Resende G.G., de Oliveira P.G., Xavier R.M., de Andrade M.V.M., Kakehasi A.M. (2023). Morphofunctional Analysis of Fibroblast-like Synoviocytes in Human Rheumatoid Arthritis and Mouse Collagen-Induced Arthritis. Adv. Rheumatol..

[183] Machado C.R.L., Resende G.G., Macedo R.B.V., do Nascimento V.C., Branco A.S., Kakehasi A.M., Andrade M.V. (2019). Fibroblast-like Synoviocytes from Fluid and Synovial Membrane from Primary Osteoarthritis Demonstrate Similar Production of Interleukin 6, and Metalloproteinases 1 and 3. Clin. Exp. Rheumatol..

[184] Ma W., Tang F., Xiao L., Han S., Yao X., Zhang Q., Zhou J., Wang Y., Zhou J. (2022). miR-205-5p in Exosomes Divided from Chondrogenic Mesenchymal Stem Cells Alleviated Rheumatoid Arthritis via Regulating MDM2 in Fibroblast-like Synoviocytes. J. Musculoskelet. Neuronal Interact..

[185] Xu W., Liu X., Qu W., Wang X., Su H., Li W., Cheng Y. (2022). Exosomes Derived from Fibrinogen-like Protein 1-Overexpressing Bone Marrow-Derived Mesenchymal Stem Cells Ameliorates Rheumatoid Arthritis. Bioengineered.

[186] Ostojic M., Zevrnja A., Vukojevic K., Soljic V. (2021). Immunofluorescence Analysis of NF-kB and iNOS Expression in Different Cell Populations during Early and Advanced Knee Osteoarthritis. Int. J. Mol. Sci..

[187] Scanzello C.R. (2017). Role of Low-Grade Inflammation in Osteoarthritis. Curr. Opin. Rheumatol..

[188] Teo K.Y.W., Zhang S., Loh J.T., Lai R.C., Hey H.W.D., Lam K.-P., Lim S.K., Toh W.S. (2023). Mesenchymal Stromal Cell Exosomes Mediate M2-like Macrophage Polarization through CD73/Ecto-5′-Nucleotidase Activity. Pharmaceutics.

[189] Lepetsos P., Papavassiliou A.G. (2016). ROS/Oxidative Stress Signaling in Osteoarthritis. Biochim. Biophys. Acta.

[190] Li X., Wang Y., Cai Z., Zhou Q., Li L., Fu P. (2021). Exosomes from Human Umbilical Cord Mesenchymal Stem Cells Inhibit ROS Production and Cell Apoptosis in Human Articular Chondrocytes via the miR-100-5p/NOX4 Axis. Cell Biol. Int..

[191] Zhou H., Shen X., Yan C., Xiong W., Ma Z., Tan Z., Wang J., Li Y., Liu J., Duan A. (2022). Extracellular Vesicles Derived from Human Umbilical Cord Mesenchymal Stem Cells Alleviate Osteoarthritis of the Knee in Mice Model by Interacting with METTL3 to Reduce m6A of NLRP3 in Macrophage. Stem Cell Res. Ther..

[192] Huleihel L., Hussey G.S., Naranjo J.D., Zhang L., Dziki J.L., Turner N.J., Stolz D.B., Badylak S.F. (2016). Matrix-Bound Nanovesicles within ECM Bioscaffolds. Sci. Adv..

[193] Peshkova M., Korneev A., Revokatova D., Smirnova O., Klyucherev T., Shender V., Arapidi G., Kosheleva N., Timashev P. (2024). Four Sides to the Story: A Proteomic Comparison of Liquid-Phase and Matrix-Bound Extracellular Vesicles in 2D and 3D Cell Cultures. Proteomics.

[194] Huleihel L., Bartolacci J.G., Dziki J.L., Vorobyov T., Arnold B., Scarritt M.E., Pineda Molina C., LoPresti S.T., Brown B.N., Naranjo J.D. (2017). Matrix-Bound Nanovesicles Recapitulate Extracellular Matrix Effects on Macrophage Phenotype. Tissue Eng. Part A.

[195] Crum R.J., Hall K., Molina C.P., Hussey G.S., Graham E., Li H., Badylak S.F. (2022). Immunomodulatory Matrix-Bound Nanovesicles Mitigate Acute and Chronic Pristane-Induced Rheumatoid Arthritis. NPJ Regen. Med..

[196] Kim H., Back J.H., Han G., Lee S.J., Park Y.E., Gu M.B., Yang Y., Lee J.E., Kim S.H. (2022). Extracellular Vesicle-Guided in Situ Reprogramming of Synovial Macrophages for the Treatment of Rheumatoid Arthritis. Biomaterials.

[197] Zhu D., Tian J., Wu X., Li M., Tang X., Rui K., Guo H., Ma J., Xu H., Wang S. (2019). G-MDSC-Derived Exosomes Attenuate Collagen-Induced Arthritis by Impairing Th1 and Th17 Cell Responses. Biochim. Biophys. Acta Mol. Basis Dis..

[198] Headland S.E., Jones H.R., Norling L.V., Kim A., Souza P.R., Corsiero E., Gil C.D., Nerviani A., Dell’Accio F., Pitzalis C. (2015). Neutrophil-Derived Microvesicles Enter Cartilage and Protect the Joint in Inflammatory Arthritis. Sci. Transl. Med..

[199] Da-Wa Z.X., Jun M., Chao-Zheng L., Sen-Lin Y., Chuan L., De-Chun L., Zu-Nan D., Hong-Tao Z., Shu-Qing W., Xian-Wei P. (2021). Exosomes Derived from M2 Macrophages Exert a Therapeutic Effect via Inhibition of the PI3K/AKT/mTOR Pathway in Rats with Knee Osteoarthritic. BioMed Res. Int..

[200] Bai J., Zhang Y., Zheng X., Huang M., Cheng W., Shan H., Gao X., Zhang M., Sheng L., Dai J. (2020). LncRNA MM2P-Induced, Exosome-Mediated Transfer of Sox9 from Monocyte-Derived Cells Modulates Primary Chondrocytes. Cell Death Dis..

[201] Qin L., Yang J., Su X., Li X., Lei Y., Dong L., Chen H., Chen C., Zhao C., Zhang H. (2023). The miR-21-5p Enriched in the Apoptotic Bodies of M2 Macrophage-Derived Extracellular Vesicles Alleviates Osteoarthritis by Changing Macrophage Phenotype. Genes Dis..

[202] Rhys H.I., Dell’Accio F., Pitzalis C., Moore A., Norling L.V., Perretti M. (2018). Neutrophil Microvesicles from Healthy Control and Rheumatoid Arthritis Patients Prevent the Inflammatory Activation of Macrophages. eBioMedicine.

[203] Feng D., Zhao W.-L., Ye Y.-Y., Bai X.-C., Liu R.-Q., Chang L.-F., Zhou Q., Sui S.-F. (2010). Cellular Internalization of Exosomes Occurs through Phagocytosis. Traffic.

[204] Xu X., Liang Y., Li X., Ouyang K., Wang M., Cao T., Li W., Liu J., Xiong J., Li B. (2021). Exosome-Mediated Delivery of Kartogenin for Chondrogenesis of Synovial Fluid-Derived Mesenchymal Stem Cells and Cartilage Regeneration. Biomaterials.

[205] You D.G., Lim G.T., Kwon S., Um W., Oh B.H., Song S.H., Lee J., Jo D.-G., Cho Y.W., Park J.H. (2021). Metabolically Engineered Stem Cell-Derived Exosomes to Regulate Macrophage Heterogeneity in Rheumatoid Arthritis. Sci. Adv..

[206] Pan Z., Sun W., Chen Y., Tang H., Lin W., Chen J., Chen C. (2022). Extracellular Vesicles in Tissue Engineering: Biology and Engineered Strategy. Adv. Healthc. Mater..

[207] Lee E.S., Sul J.H., Shin J.M., Shin S., Lee J.A., Kim H.K., Cho Y., Ko H., Son S., Lee J. (2021). Reactive Oxygen Species-Responsive Dendritic Cell-Derived Exosomes for Rheumatoid Arthritis. Acta Biomater..

[208] Duan A., Shen K., Li B., Li C., Zhou H., Kong R., Shao Y., Qin J., Yuan T., Ji J. (2021). Extracellular Vesicles Derived from LPS-Preconditioned Human Synovial Mesenchymal Stem Cells Inhibit Extracellular Matrix Degradation and Prevent Osteoarthritis of the Knee in a Mouse Model. Stem Cell Res. Ther..

[209] Rong Y., Zhang J., Jiang D., Ji C., Liu W., Wang J., Ge X., Tang P., Yu S., Cui W. (2021). Hypoxic Pretreatment of Small Extracellular Vesicles Mediates Cartilage Repair in Osteoarthritis by Delivering miR-216a-5p. Acta Biomater..

[210] Ragni E., Perucca Orfei C., De Luca P., Colombini A., Viganò M., de Girolamo L. (2020). Secreted Factors and EV-miRNAs Orchestrate the Healing Capacity of Adipose Mesenchymal Stem Cells for the Treatment of Knee Osteoarthritis. Int. J. Mol. Sci..

[211] Yan L., Wu X. (2020). Exosomes Produced from 3D Cultures of Umbilical Cord Mesenchymal Stem Cells in a Hollow-Fiber Bioreactor Show Improved Osteochondral Regeneration Activity. Cell Biol. Toxicol..

[212] Zhang B., Tian X., Qu Z., Hao J., Zhang W. (2022). Hypoxia-Preconditioned Extracellular Vesicles from Mesenchymal Stem Cells Improve Cartilage Repair in Osteoarthritis. Membranes.

[213] Saller M.M., Prall W.C., Docheva D., Schönitzer V., Popov T., Anz D., Clausen-Schaumann H., Mutschler W., Volkmer E., Schieker M. (2012). Increased Stemness and Migration of Human Mesenchymal Stem Cells in Hypoxia Is Associated with Altered Integrin Expression. Biochem. Biophys. Res. Commun..

[214] Gonzalez-King H., García N.A., Ontoria-Oviedo I., Ciria M., Montero J.A., Sepúlveda P. (2017). Hypoxia Inducible Factor-1α Potentiates Jagged 1-Mediated Angiogenesis by Mesenchymal Stem Cell-Derived Exosomes. Stem Cells.

[215] Gorin C., Rochefort G.Y., Bascetin R., Ying H., Lesieur J., Sadoine J., Beckouche N., Berndt S., Novais A., Lesage M. (2016). Priming Dental Pulp Stem Cells With Fibroblast Growth Factor-2 Increases Angiogenesis of Implanted Tissue-Engineered Constructs Through Hepatocyte Growth Factor and Vascular Endothelial Growth Factor Secretion. Stem Cells Transl. Med..

[216] Rocha S., Carvalho J., Oliveira P., Voglstaetter M., Schvartz D., Thomsen A.R., Walter N., Khanduri R., Sanchez J.-C., Keller A. (2019). 3D Cellular Architecture Affects MicroRNA and Protein Cargo of Extracellular Vesicles. Adv. Sci..

[217] Haraszti R.A., Miller R., Stoppato M., Sere Y.Y., Coles A., Didiot M.-C., Wollacott R., Sapp E., Dubuke M.L., Li X. (2018). Exosomes Produced from 3D Cultures of MSCs by Tangential Flow Filtration Show Higher Yield and Improved Activity. Mol. Ther. J. Am. Soc. Gene Ther..

[218] Kim H., Lee M.J., Bae E.-H., Ryu J.S., Kaur G., Kim H.J., Kim J.Y., Barreda H., Jung S.Y., Choi J.M. (2020). Comprehensive Molecular Profiles of Functionally Effective MSC-Derived Extracellular Vesicles in Immunomodulation. Mol. Ther. J. Am. Soc. Gene Ther..

[219] Rashedi I., Gómez-Aristizábal A., Wang X.-H., Viswanathan S., Keating A. (2017). TLR3 or TLR4 Activation Enhances Mesenchymal Stromal Cell-Mediated Treg Induction via Notch Signaling. Stem Cells.

[220] Cheng A., Choi D., Lora M., Shum-Tim D., Rak J., Colmegna I. (2020). Human Multipotent Mesenchymal Stromal Cells Cytokine Priming Promotes RAB27B-Regulated Secretion of Small Extracellular Vesicles with Immunomodulatory Cargo. Stem Cell Res. Ther..

[221] Domenis R., Cifù A., Quaglia S., Pistis C., Moretti M., Vicario A., Parodi P.C., Fabris M., Niazi K.R., Soon-Shiong P. (2018). Pro Inflammatory Stimuli Enhance the Immunosuppressive Functions of Adipose Mesenchymal Stem Cells-Derived Exosomes. Sci. Rep..

[222] Li S., Stöckl S., Lukas C., Herrmann M., Brochhausen C., König M.A., Johnstone B., Grässel S. (2021). Curcumin-Primed Human BMSC-Derived Extracellular Vesicles Reverse IL-1β-Induced Catabolic Responses of OA Chondrocytes by Upregulating miR-126-3p. Stem Cell Res. Ther..

[223] Wang R., Xu B. (2021). TGF-Β1-Modified MSC-Derived Exosomal miR-135b Attenuates Cartilage Injury via Promoting M2 Synovial Macrophage Polarization by Targeting MAPK6. Cell Tissue Res..

[224] Kim S.-H., Lechman E.R., Bianco N., Menon R., Keravala A., Nash J., Mi Z., Watkins S.C., Gambotto A., Robbins P.D. (2005). Exosomes Derived from IL-10-Treated Dendritic Cells Can Suppress Inflammation and Collagen-Induced Arthritis. J. Immunol..

[225] Zhang S., Jin Z. (2022). Bone Mesenchymal Stem Cell-Derived Extracellular Vesicles Containing Long Noncoding RNA NEAT1 Relieve Osteoarthritis. Oxid. Med. Cell. Longev..

[226] Sage E.K., Thakrar R.M., Janes S.M. (2016). Genetically Modified Mesenchymal Stromal Cells in Cancer Therapy. Cytotherapy.

[227] Oggu G.S., Sasikumar S., Reddy N., Ella K.K.R., Rao C.M., Bokara K.K. (2017). Gene Delivery Approaches for Mesenchymal Stem Cell Therapy: Strategies to Increase Efficiency and Specificity. Stem Cell Rev. Rep..

[228] Doi K., Takeuchi Y. (2015). Gene therapy using retrovirus vectors: Vector development and biosafety at clinical trials. Uirusu.

[229] Ricks D.M., Kutner R., Zhang X.-Y., Welsh D.A., Reiser J. (2008). Optimized Lentiviral Transduction of Mouse Bone Marrow-Derived Mesenchymal Stem Cells. Stem Cells Dev..

[230] Zaiss A.-K., Liu Q., Bowen G.P., Wong N.C.W., Bartlett J.S., Muruve D.A. (2002). Differential Activation of Innate Immune Responses by Adenovirus and Adeno-Associated Virus Vectors. J. Virol..

[231] Shen T., Alvarez-Garcia O., Li Y., Olmer M., Lotz M.K. (2017). Suppression of Sestrins in Aging and Osteoarthritic Cartilage: Dysfunction of an Important Stress Defense Mechanism. Osteoarthr. Cartil..

[232] Meng H.-Y., Chen L.-Q., Chen L.-H. (2020). The Inhibition by Human MSCs-Derived miRNA-124a Overexpression Exosomes in the Proliferation and Migration of Rheumatoid Arthritis-Related Fibroblast-like Synoviocyte Cell. BMC Musculoskelet. Disord..

[233] Wang Y., Dai L., Wu H., Zhang Z.-R., Wang W.-Y., Fu J., Deng R., Li F., Dai X.-J., Zhan X. (2018). Novel Anti-Inflammatory Target of Geniposide: Inhibiting Itgβ1/Ras-Erk1/2 Signal Pathway via the miRNA-124a in Rheumatoid Arthritis Synovial Fibroblasts. Int. Immunopharmacol..

[234] Bianco N.R., Kim S.H., Ruffner M.A., Robbins P.D. (2009). Therapeutic Effect of Exosomes from Indoleamine 2,3-Dioxygenase-Positive Dendritic Cells in Collagen-Induced Arthritis and Delayed-Type Hypersensitivity Disease Models. Arthritis Rheum..

[235] Yin H., Kanasty R.L., Eltoukhy A.A., Vegas A.J., Dorkin J.R., Anderson D.G. (2014). Non-Viral Vectors for Gene-Based Therapy. Nat. Rev. Genet..

[236] Hamann A., Nguyen A., Pannier A.K. (2019). Nucleic Acid Delivery to Mesenchymal Stem Cells: A Review of Nonviral Methods and Applications. J. Biol. Eng..

[237] Park J.S., Na K., Woo D.G., Yang H.N., Kim J.M., Kim J.H., Chung H.-M., Park K.-H. (2010). Non-Viral Gene Delivery of DNA Polyplexed with Nanoparticles Transfected into Human Mesenchymal Stem Cells. Biomaterials.

[238] Liu W., Liu A., Li X., Sun Z., Sun Z., Liu Y., Wang G., Huang D., Xiong H., Yu S. (2023). Dual-Engineered Cartilage-Targeting Extracellular Vesicles Derived from Mesenchymal Stem Cells Enhance Osteoarthritis Treatment via miR-223/NLRP3/Pyroptosis Axis: Toward a Precision Therapy. Bioact. Mater..

[239] Chang L., Kan L. (2021). Mesenchymal Stem Cell-Originated Exosomal Circular RNA circFBXW7 Attenuates Cell Proliferation, Migration and Inflammation of Fibroblast-Like Synoviocytes by Targeting miR-216a-3p/HDAC4 in Rheumatoid Arthritis. J. Inflamm. Res..

[240] Yan L., Liu G., Wu X. (2021). The Umbilical Cord Mesenchymal Stem Cell-Derived Exosomal lncRNA H19 Improves Osteochondral Activity through miR-29b-3p/FoxO3 Axis. Clin. Transl. Med..

[241] Saliminejad K., Khorram Khorshid H.R., Soleymani Fard S., Ghaffari S.H. (2019). An Overview of microRNAs: Biology, Functions, Therapeutics, and Analysis Methods. J. Cell. Physiol..

[242] Lu T.X., Rothenberg M.E. (2018). MicroRNA. J. Allergy Clin. Immunol..

[243] Nygaard G., Firestein G.S. (2020). Restoring Synovial Homeostasis in Rheumatoid Arthritis by Targeting Fibroblast-like Synoviocytes. Nat. Rev. Rheumatol..

[244] Wang Y., Wu H., Deng R. (2021). Angiogenesis as a Potential Treatment Strategy for Rheumatoid Arthritis. Eur. J. Pharmacol..

[245] Li J., Zhang Y., Liu Y., Dai X., Li W., Cai X., Yin Y., Wang Q., Xue Y., Wang C. (2013). Microvesicle-Mediated Transfer of microRNA-150 from Monocytes to Endothelial Cells Promotes Angiogenesis. J. Biol. Chem..

[246] Chen Z., Wang H., Xia Y., Yan F., Lu Y. (2018). Therapeutic Potential of Mesenchymal Cell-Derived miRNA-150-5p-Expressing Exosomes in Rheumatoid Arthritis Mediated by the Modulation of MMP14 and VEGF. J. Immunol..

[247] Lin Z., Li W., Wang Y., Lang X., Sun W., Zhu X., Bian R., Ma Y., Wei X., Zhang J. (2023). SMSCs-Derived sEV Overexpressing miR-433-3p Inhibits Angiogenesis Induced by sEV Released from Synoviocytes under Triggering of Ferroptosis. Int. Immunopharmacol..

[248] Wang Z., Yan K., Ge G., Zhang D., Bai J., Guo X., Zhou J., Xu T., Xu M., Long X. (2021). Exosomes Derived from miR-155-5p-Overexpressing Synovial Mesenchymal Stem Cells Prevent Osteoarthritis via Enhancing Proliferation and Migration, Attenuating Apoptosis, and Modulating Extracellular Matrix Secretion in Chondrocytes. Cell Biol. Toxicol..

[249] Chen D., Kim D.J., Shen J., Zou Z., O’Keefe R.J. (2020). Runx2 Plays a Central Role in Osteoarthritis Development. J. Orthop. Transl..

[250] Huang Y., Zhang X., Zhan J., Yan Z., Chen D., Xue X., Pan X. (2021). Bone Marrow Mesenchymal Stem Cell-Derived Exosomal miR-206 Promotes Osteoblast Proliferation and Differentiation in Osteoarthritis by Reducing Elf3. J. Cell. Mol. Med..

[251] Wondimu E.B., Culley K.L., Quinn J., Chang J., Dragomir C.L., Plumb D.A., Goldring M.B., Otero M. (2018). Elf3 Contributes to Cartilage Degradation in Vivo in a Surgical Model of Post-Traumatic Osteoarthritis. Sci. Rep..

[252] Conde J., Otero M., Scotece M., Abella V., Gómez R., López V., Pino J., Mera A., Goldring M.B., Gualillo O. (2018). E74-Like Factor (ELF3) and Leptin, a Novel Loop Between Obesity and Inflammation Perpetuating a Pro-Catabolic State in Cartilage. Cell. Physiol. Biochem. Int. J. Exp. Cell. Physiol. Biochem. Pharmacol..

[253] Li S., Liu J., Liu S., Jiao W., Wang X. (2021). Mesenchymal Stem Cell-Derived Extracellular Vesicles Prevent the Development of Osteoarthritis via the circHIPK3/miR-124-3p/MYH9 Axis. J. Nanobiotechnol..

[254] Liu D., Liang Y.-H., Yang Y.-T., He M., Cai Z.-J., Xiao W.-F., Li Y.-S. (2021). Circular RNA in Osteoarthritis: An Updated Insight into the Pathophysiology and Therapeutics. Am. J. Transl. Res..

[255] Kristensen L.S., Andersen M.S., Stagsted L.V.W., Ebbesen K.K., Hansen T.B., Kjems J. (2019). The Biogenesis, Biology and Characterization of Circular RNAs. Nat. Rev. Genet..

[256] Mao G., Xu Y., Long D., Sun H., Li H., Xin R., Zhang Z., Li Z., Yang Z., Kang Y. (2021). Exosome-Transported circRNA_0001236 Enhances Chondrogenesis and Suppress Cartilage Degradation via the miR-3677-3p/Sox9 Axis. Stem Cell Res. Ther..

[257] Zhang J., Zhang Y., Ma Y., Luo L., Chu M., Zhang Z. (2021). Therapeutic Potential of Exosomal circRNA Derived from Synovial Mesenchymal Cells via Targeting circEDIL3/miR-485-3p/PIAS3/STAT3/VEGF Functional Module in Rheumatoid Arthritis. Int. J. Nanomed..

[258] Yang Q., Yao Y., Zhao D., Zou H., Lai C., Xiang G., Wang G., Luo L., Shi Y., Li Y. (2021). LncRNA H19 Secreted by Umbilical Cord Blood Mesenchymal Stem Cells through microRNA-29a-3p/FOS Axis for Central Sensitization of Pain in Advanced Osteoarthritis. Am. J. Transl. Res..

[259] Su Y., Liu Y., Ma C., Guan C., Ma X., Meng S. (2021). Mesenchymal Stem Cell-Originated Exosomal lncRNA HAND2-AS1 Impairs Rheumatoid Arthritis Fibroblast-like Synoviocyte Activation through miR-143-3p/TNFAIP3/NF-κB Pathway. J. Orthop. Surg..

[260] Wang Q., Yu J., Kadungure T., Beyene J., Zhang H., Lu Q. (2018). ARMMs as a Versatile Platform for Intracellular Delivery of Macromolecules. Nat. Commun..

[261] Kim S.H., Bianco N.R., Shufesky W.J., Morelli A.E., Robbins P.D. (2007). Effective Treatment of Inflammatory Disease Models with Exosomes Derived from Dendritic Cells Genetically Modified to Express IL-4. J. Immunol..

[262] Xie A., Xue J., Wang Y., Yang C., Xu M., Jiang Y. (2022). Kartogenin Induced Adipose-Derived Stem Cell Exosomes Enhance the Chondrogenic Differentiation Ability of Adipose-Derived Stem Cells. Dis. Mark..

[263] Junxu L., Yuanbo W., Yanuan H., Biaobing Y. (2020). Recent Advances and Applications of Kartogenin in Cartilage Regeneration and Repair. Chin. J. Tissue Eng. Res..

[264] Li H., Feng Y., Zheng X., Jia M., Mei Z., Wang Y., Zhang Z., Zhou M., Li C. (2022). M2-Type Exosomes Nanoparticles for Rheumatoid Arthritis Therapy via Macrophage Re-Polarization. J. Control. Release.

[265] Mao G., Zhang Z., Hu S., Zhang Z., Chang Z., Huang Z., Liao W., Kang Y. (2018). Exosomes Derived from miR-92a-3p-Overexpressing Human Mesenchymal Stem Cells Enhance Chondrogenesis and Suppress Cartilage Degradation via Targeting WNT5A. Stem Cell Res. Ther..

[266] Feng K., Xie X., Yuan J., Gong L., Zhu Z., Zhang J., Li H., Yang Y., Wang Y. (2021). Reversing the Surface Charge of MSC-Derived Small Extracellular Vesicles by εPL-PEG-DSPE for Enhanced Osteoarthritis Treatment. J. Extracell. Vesicles.

[267] Wu J., Kuang L., Chen C., Yang J., Zeng W.-N., Li T., Chen H., Huang S., Fu Z., Li J. (2019). miR-100-5p-Abundant Exosomes Derived from Infrapatellar Fat Pad MSCs Protect Articular Cartilage and Ameliorate Gait Abnormalities via Inhibition of mTOR in Osteoarthritis. Biomaterials.

[268] Tao Y., Zhou J., Wang Z., Tao H., Bai J., Ge G., Li W., Zhang W., Hao Y., Yang X. (2021). Human Bone Mesenchymal Stem Cells-Derived Exosomal miRNA-361-5p Alleviates Osteoarthritis by Downregulating DDX20 and Inactivating the NF-κB Signaling Pathway. Bioorg. Chem..

[269] Wu H., Zhou X., Wang X., Cheng W., Hu X., Wang Y., Luo B., Huang W., Gu J. (2021). miR-34a in Extracellular Vesicles from Bone Marrow Mesenchymal Stem Cells Reduces Rheumatoid Arthritis Inflammation via the Cyclin I/ATM/ATR/P53 Axis. J. Cell. Mol. Med..

[270] Meng Q., Qiu B. (2020). Exosomal MicroRNA-320a Derived From Mesenchymal Stem Cells Regulates Rheumatoid Arthritis Fibroblast-Like Synoviocyte Activation by Suppressing CXCL9 Expression. Front. Physiol..

[271] Jin Z., Ren J., Qi S. (2020). Exosomal miR-9-5p Secreted by Bone Marrow-Derived Mesenchymal Stem Cells Alleviates Osteoarthritis by Inhibiting Syndecan-1. Cell Tissue Res..

[272] Wiklander O.P.B., Nordin J.Z., O’Loughlin A., Gustafsson Y., Corso G., Mäger I., Vader P., Lee Y., Sork H., Seow Y. (2015). Extracellular Vesicle in Vivo Biodistribution Is Determined by Cell Source, Route of Administration and Targeting. J. Extracell. Vesicles.

[273] Smyth T., Kullberg M., Malik N., Smith-Jones P., Graner M.W., Anchordoquy T.J. (2015). Biodistribution and Delivery Efficiency of Unmodified Tumor-Derived Exosomes. J. Control. Release.

[274] Lai C.P., Mardini O., Ericsson M., Prabhakar S., Maguire C., Chen J.W., Tannous B.A., Breakefield X.O. (2014). Dynamic Biodistribution of Extracellular Vesicles in Vivo Using a Multimodal Imaging Reporter. ACS Nano.

[275] Cheng H., Byrska-Bishop M., Zhang C.T., Kastrup C.J., Hwang N.S., Tai A.K., Lee W.W., Xu X., Nahrendorf M., Langer R. (2012). Stem Cell Membrane Engineering for Cell Rolling Using Peptide Conjugation and Tuning of Cell-Selectin Interaction Kinetics. Biomaterials.

[276] Mura S., Nicolas J., Couvreur P. (2013). Stimuli-Responsive Nanocarriers for Drug Delivery. Nat. Mater..

[277] Agatemor C., Buettner M.J., Ariss R., Muthiah K., Saeui C.T., Yarema K.J. (2019). Exploiting Metabolic Glycoengineering to Advance Healthcare. Nat. Rev. Chem..

[278] Nagasundaram M., Horstkorte R., Gnanapragassam V.S. (2020). Sialic Acid Metabolic Engineering of Breast Cancer Cells Interferes with Adhesion and Migration. Molecules.

[279] Li S., Yu B., Wang J., Zheng Y., Zhang H., Walker M.J., Yuan Z., Zhu H., Zhang J., Wang P.G. (2018). Biomarker-Based Metabolic Labeling for Redirected and Enhanced Immune Response. ACS Chem. Biol..

[280] Xia Y., Rao L., Yao H., Wang Z., Ning P., Chen X. (2020). Engineering Macrophages for Cancer Immunotherapy and Drug Delivery. Adv. Mater..

[281] Rao L., Zhao S.-K., Wen C., Tian R., Lin L., Cai B., Sun Y., Kang F., Yang Z., He L. (2020). Activating Macrophage-Mediated Cancer Immunotherapy by Genetically Edited Nanoparticles. Adv. Mater..

[282] Yoon H.Y., Koo H., Kim K., Kwon I.C. (2017). Molecular Imaging Based on Metabolic Glycoengineering and Bioorthogonal Click Chemistry. Biomaterials.

[283] Ahern B.J., Parvizi J., Boston R., Schaer T.P. (2009). Preclinical Animal Models in Single Site Cartilage Defect Testing: A Systematic Review. Osteoarthr. Cartil..

[284] Liang Y., Xu X., Li X., Xiong J., Li B., Duan L., Wang D., Xia J. (2020). Chondrocyte-Targeted MicroRNA Delivery by Engineered Exosomes toward a Cell-Free Osteoarthritis Therapy. ACS Appl. Mater. Interfaces.

[285] Takahashi Y., Nishikawa M., Shinotsuka H., Matsui Y., Ohara S., Imai T., Takakura Y. (2013). Visualization and in Vivo Tracking of the Exosomes of Murine Melanoma B16-BL6 Cells in Mice after Intravenous Injection. J. Biotechnol..

[286] Ranjan P., Colin K., Dutta R.K., Verma S.K. (2023). Challenges and Future Scope of Exosomes in the Treatment of Cardiovascular Diseases. J. Physiol..

[287] Chen P., Zheng L., Wang Y., Tao M., Xie Z., Xia C., Gu C., Chen J., Qiu P., Mei S. (2019). Desktop-Stereolithography 3D Printing of a Radially Oriented Extracellular Matrix/Mesenchymal Stem Cell Exosome Bioink for Osteochondral Defect Regeneration. Theranostics.

[288] Zhao C., Song W., Ma J., Wang N. (2022). Macrophage-Derived Hybrid Exosome-Mimic Nanovesicles Loaded with Black Phosphorus for Multimodal Rheumatoid Arthritis Therapy. Biomater. Sci..

[289] Quiñonez-Flores C.M., González-Chávez S.A., Del Río Nájera D., Pacheco-Tena C. (2016). Oxidative Stress Relevance in the Pathogenesis of the Rheumatoid Arthritis: A Systematic Review. BioMed Res. Int..

[290] Rambhia K.J., Ma P.X. (2015). Controlled Drug Release for Tissue Engineering. J. Control. Release.

[291] Jiang S., Tian G., Yang Z., Gao X., Wang F., Li J., Tian Z., Huang B., Wei F., Sang X. (2021). Enhancement of Acellular Cartilage Matrix Scaffold by Wharton’s Jelly Mesenchymal Stem Cell-Derived Exosomes to Promote Osteochondral Regeneration. Bioact. Mater..

[292] Bao W., Li M., Yang Y., Wan Y., Wang X., Bi N., Li C. (2020). Advancements and Frontiers in the High Performance of Natural Hydrogels for Cartilage Tissue Engineering. Front. Chem..

[293] Lenzini S., Bargi R., Chung G., Shin J.-W. (2020). Matrix Mechanics and Water Permeation Regulate Extracellular Vesicle Transport. Nat. Nanotechnol..

[294] Huang J., Xiong J., Yang L., Zhang J., Sun S., Liang Y. (2021). Cell-Free Exosome-Laden Scaffolds for Tissue Repair. Nanoscale.

[295] Pang L., Jin H., Lu Z., Xie F., Shen H., Li X., Zhang X., Jiang X., Wu L., Zhang M. (2023). Treatment with Mesenchymal Stem Cell-Derived Nanovesicle-Containing Gelatin Methacryloyl Hydrogels Alleviates Osteoarthritis by Modulating Chondrogenesis and Macrophage Polarization. Adv. Healthc. Mater..

[296] Zhang H., Huang J., Alahdal M. (2023). Exosomes Loaded with Chondrogenic Stimuli Agents Combined with 3D Bioprinting Hydrogel in the Treatment of Osteoarthritis and Cartilage Degeneration. Biomed. Pharmacother..

[297] Wang Y., Qin J.-Z., Xie C.-Y., Peng X.-Z., Wang J.-H., Wang S.-J. (2024). Kartogenin-Loaded Exosomes Derived from Bone Marrow Mesenchymal Stem Cells Enhance Chondrogenesis and Expedite Tendon Enthesis Healing in a Rat Model of Rotator Cuff Injury. Am. J. Sports Med..

[298] Cai J., Xu J., Ye Z., Wang L., Zheng T., Zhang T., Li Y., Jiang J., Zhao J. (2023). Exosomes Derived from Kartogenin-Preconditioned Mesenchymal Stem Cells Promote Cartilage Formation and Collagen Maturation for Enthesis Regeneration in a Rat Model of Chronic Rotator Cuff Tear. Am. J. Sports Med..

[299] Liu X., Yang Y., Li Y., Niu X., Zhao B., Wang Y., Bao C., Xie Z., Lin Q., Zhu L. (2017). Integration of Stem Cell-Derived Exosomes with in Situ Hydrogel Glue as a Promising Tissue Patch for Articular Cartilage Regeneration. Nanoscale.

[300] Woo C.H., Kim H.K., Jung G.Y., Jung Y.J., Lee K.S., Yun Y.E., Han J., Lee J., Kim W.S., Choi J.S. (2020). Small Extracellular Vesicles from Human Adipose-Derived Stem Cells Attenuate Cartilage Degeneration. J. Extracell. Vesicles.

[301] Lopez-Santalla M., Fernandez-Perez R., Garin M.I. (2020). Mesenchymal Stem/Stromal Cells for Rheumatoid Arthritis Treatment: An Update on Clinical Applications. Cells.

[302] Pers Y.-M., Rackwitz L., Ferreira R., Pullig O., Delfour C., Barry F., Sensebe L., Casteilla L., Fleury S., Bourin P. (2016). Adipose Mesenchymal Stromal Cell-Based Therapy for Severe Osteoarthritis of the Knee: A Phase I Dose-Escalation Trial. Stem Cells Transl. Med..

[303] Orozco L., Munar A., Soler R., Alberca M., Soler F., Huguet M., Sentís J., Sánchez A., García-Sancho J. (2013). Treatment of Knee Osteoarthritis with Autologous Mesenchymal Stem Cells: A Pilot Study. Transplantation.

[304] Álvaro-Gracia J.M., Jover J.A., García-Vicuña R., Carreño L., Alonso A., Marsal S., Blanco F., Martínez-Taboada V.M., Taylor P., Martín-Martín C. (2017). Intravenous Administration of Expanded Allogeneic Adipose-Derived Mesenchymal Stem Cells in Refractory Rheumatoid Arthritis (Cx611): Results of a Multicentre, Dose Escalation, Randomised, Single-Blind, Placebo-Controlled Phase Ib/IIa Clinical Trial. Ann. Rheum. Dis..

[305] Garay-Mendoza D., Villarreal-Martínez L., Garza-Bedolla A., Pérez-Garza D.M., Acosta-Olivo C., Vilchez-Cavazos F., Diaz-Hutchinson C., Gómez-Almaguer D., Jaime-Pérez J.C., Mancías-Guerra C. (2018). The Effect of Intra-Articular Injection of Autologous Bone Marrow Stem Cells on Pain and Knee Function in Patients with Osteoarthritis. Int. J. Rheum. Dis..

[306] Shadmanfar S., Labibzadeh N., Emadedin M., Jaroughi N., Azimian V., Mardpour S., Kakroodi F.A., Bolurieh T., Hosseini S.E., Chehrazi M. (2018). Intra-Articular Knee Implantation of Autologous Bone Marrow-Derived Mesenchymal Stromal Cells in Rheumatoid Arthritis Patients with Knee Involvement: Results of a Randomized, Triple-Blind, Placebo-Controlled Phase 1/2 Clinical Trial. Cytotherapy.

[307] Bastos R., Mathias M., Andrade R., Amaral R.J.F.C., Schott V., Balduino A., Bastos R., Miguel Oliveira J., Reis R.L., Rodeo S. (2020). Intra-Articular Injection of Culture-Expanded Mesenchymal Stem Cells with or without Addition of Platelet-Rich Plasma Is Effective in Decreasing Pain and Symptoms in Knee Osteoarthritis: A Controlled, Double-Blind Clinical Trial. Knee Surg. Sports Traumatol. Arthrosc..

[308] Copp G., Robb K.P., Viswanathan S. (2023). Culture-Expanded Mesenchymal Stromal Cell Therapy: Does It Work in Knee Osteoarthritis? A Pathway to Clinical Success. Cell. Mol. Immunol..

[309] Schioppo T., Ubiali T., Ingegnoli F., Bollati V., Caporali R. (2021). The Role of Extracellular Vesicles in Rheumatoid Arthritis: A Systematic Review. Clin. Rheumatol..

[310] Schmitz C., Alt C., Pearce D.A., Furia J.P., Maffulli N., Alt E.U. (2022). Methodological Flaws in Meta-Analyses of Clinical Studies on the Management of Knee Osteoarthritis with Stem Cells: A Systematic Review. Cells.

[311] Wiggers T.G., Winters M., Van den Boom N.A., Haisma H.J., Moen M.H. (2021). Autologous Stem Cell Therapy in Knee Osteoarthritis: A Systematic Review of Randomised Controlled Trials. Br. J. Sports Med..

[312] Carneiro D.d.C., Araújo L.T.d., Santos G.C., Damasceno P.K.F., Vieira J.L., Santos R.R.d., Barbosa J.D.V., Soares M.B.P. (2023). Clinical Trials with Mesenchymal Stem Cell Therapies for Osteoarthritis: Challenges in the Regeneration of Articular Cartilage. Int. J. Mol. Sci..

[313] Sarsenova M., Issabekova A., Abisheva S., Rutskaya-Moroshan K., Ogay V., Saparov A. (2021). Mesenchymal Stem Cell-Based Therapy for Rheumatoid Arthritis. Int. J. Mol. Sci..

[314] Chu C.R., Szczodry M., Bruno S. (2010). Animal Models for Cartilage Regeneration and Repair. Tissue Eng. Part B Rev..

[315] Haraszti R.A., Miller R., Dubuke M.L., Rockwell H.E., Coles A.H., Sapp E., Didiot M.-C., Echeverria D., Stoppato M., Sere Y.Y. (2019). Serum Deprivation of Mesenchymal Stem Cells Improves Exosome Activity and Alters Lipid and Protein Composition. iScience.

[316] Otsuru S., Hofmann T.J., Raman P., Olson T.S., Guess A.J., Dominici M., Horwitz E.M. (2015). Genomic and Functional Comparison of Mesenchymal Stromal Cells Prepared Using Two Isolation Methods. Cytotherapy.

[317] Toh W.S., Brittberg M., Farr J., Foldager C.B., Gomoll A.H., Hui J.H.P., Richardson J.B., Roberts S., Spector M. (2016). Cellular Senescence in Aging and Osteoarthritis. Acta Orthop..

[318] Siegel G., Kluba T., Hermanutz-Klein U., Bieback K., Northoff H., Schäfer R. (2013). Phenotype, Donor Age and Gender Affect Function of Human Bone Marrow-Derived Mesenchymal Stromal Cells. BMC Med..

[319] McKee C., Chaudhry G.R. (2017). Advances and Challenges in Stem Cell Culture. Colloids Surf. B Biointerfaces.

[320] Liu Z., Zhuang Y., Fang L., Yuan C., Wang X., Lin K. (2022). Breakthrough of Extracellular Vesicles in Pathogenesis, Diagnosis and Treatment of Osteoarthritis. Bioact. Mater..

[321] Coppin L., Sokal E., Stéphenne X. (2019). Thrombogenic Risk Induced by Intravascular Mesenchymal Stem Cell Therapy: Current Status and Future Perspectives. Cells.

[322] Lee R.H., Pulin A.A., Seo M.J., Kota D.J., Ylostalo J., Larson B.L., Semprun-Prieto L., Delafontaine P., Prockop D.J. (2009). Intravenous hMSCs Improve Myocardial Infarction in Mice Because Cells Embolized in Lung Are Activated to Secrete the Anti-Inflammatory Protein TSG-6. Cell Stem Cell.

[323] Phinney D.G., Pittenger M.F. (2017). Concise Review: MSC-Derived Exosomes for Cell-Free Therapy. Stem Cells.

[324] Théry C., Witwer K.W., Aikawa E., Alcaraz M.J., Anderson J.D., Andriantsitohaina R., Antoniou A., Arab T., Archer F., Atkin-Smith G.K. (2018). Minimal Information for Studies of Extracellular Vesicles 2018 (MISEV2018): A Position Statement of the International Society for Extracellular Vesicles and Update of the MISEV2014 Guidelines. J. Extracell. Vesicles.

[325] Jeyaram A., Jay S.M. (2017). Preservation and Storage Stability of Extracellular Vesicles for Therapeutic Applications. AAPS J..

[326] Wiklander O.P.B., Brennan M.Á., Lötvall J., Breakefield X.O., El Andaloussi S. (2019). Advances in Therapeutic Applications of Extracellular Vesicles. Sci. Transl. Med..

[327] Moll G., Alm J.J., Davies L.C., von Bahr L., Heldring N., Stenbeck-Funke L., Hamad O.A., Hinsch R., Ignatowicz L., Locke M. (2014). Do Cryopreserved Mesenchymal Stromal Cells Display Impaired Immunomodulatory and Therapeutic Properties?. Stem Cells.

[328] Allan D., Tieu A., Lalu M., Burger D. (2020). Mesenchymal Stromal Cell-Derived Extracellular Vesicles for Regenerative Therapy and Immune Modulation: Progress and Challenges toward Clinical Application. Stem Cells Transl. Med..

[329] Kam M., Bekkers R. (2020). Comparative Analysis of Standardized Protocols for EV Roaming.

[330] Toh W.S., Yarani R., Andaloussi S.E., Cho B.S., Choi C., Corteling R., Fougerolles A.D., Gimona M., Herz J., Khoury M. (2023). A Report on the International Society for Cell & Gene Therapy 2022 Scientific Signature Series, “Therapeutic Advances with Native and Engineered Human Extracellular Vesicles”. Cytotherapy.

[331] Claridge B., Lozano J., Poh Q.H., Greening D.W. (2021). Development of Extracellular Vesicle Therapeutics: Challenges, Considerations, and Opportunities. Front. Cell Dev. Biol..

[332] Wang C.-K., Tsai T.-H., Lee C.-H. (2024). Regulation of Exosomes as Biologic Medicines: Regulatory Challenges Faced in Exosome Development and Manufacturing Processes. Clin. Transl. Sci..

[333] U.S. Food and Drug Administration (2020). Regulatory Considerations for Human Cells, Tissues, and Cellular and Tissue-Based Products: Minimal Manipulation and Homologous Use. Guidance for Industry and Food and Drug Administration Staff.

[334] Lerussi G., Villagrasa-Araya V., Moltó-Abad M., del Toro M., Pintos-Morell G., Seras-Franzoso J., Abasolo I. (2025). Extracellular Vesicles as Tools for Crossing the Blood–Brain Barrier to Treat Lysosomal Storage Diseases. Life.

[335] Shami-shah A., Travis B.G., Walt D.R. (2023). Advances in Extracellular Vesicle Isolation Methods: A Path towards Cell-Type Specific EV Isolation. Extracell. Vesicles Circ. Nucleic Acids.

[336] Nelson B.C., Maragh S., Ghiran I.C., Jones J.C., DeRose P.C., Elsheikh E., Vreeland W.N., Wang L. (2020). Measurement and Standardization Challenges for Extracellular Vesicle Therapeutic Delivery Vectors. Nanomedicine.

[337] Momen-Heravi F. (2017). Isolation of Extracellular Vesicles by Ultracentrifugation. Methods Mol Biol..

[338] Ramirez M.I., Amorim M.G., Gadelha C., Milic I., Welsh J.A., Freitas V.M., Nawaz M., Akbar N., Couch Y., Makin L. (2018). Technical Challenges of Working with Extracellular Vesicles. Nanoscale.

[339] Cricrì G., Bellucci L., Montini G., Collino F. (2021). Urinary Extracellular Vesicles: Uncovering the Basis of the Pathological Processes in Kidney-Related Diseases. Int. J. Mol. Sci..

[340] Jones M.T., Manioci S.W., Russell A.E. (2022). Size Exclusion Chromatography for Separating Extracellular Vesicles from Conditioned Cell Culture Media. J. Vis. Exp. JoVE.

[341] Sidhom K., Obi P.O., Saleem A. (2020). A Review of Exosomal Isolation Methods: Is Size Exclusion Chromatography the Best Option?. Int. J. Mol. Sci..

[342] Paganini C., Palmiero U.C., Pocsfalvi G., Touzet N., Bongiovanni A., Arosio P. (2019). Scalable Production and Isolation of Extracellular Vesicles: Available Sources and Lessons from Current Industrial Bioprocesses. Biotechnol. J..

[343] Chernyshev V.S., Yashchenok A., Ivanov M., Silachev D.N. (2023). Filtration-Based Technologies for Isolation, Purification and Analysis of Extracellular Vesicles. Phys. Chem. Chem. Phys..

[344] Szatanek R., Baran J., Siedlar M., Baj-Krzyworzeka M. (2015). Isolation of Extracellular Vesicles: Determining the Correct Approach (Review). Int. J. Mol. Med..

[345] Mas-Bargues C., Borrás C. (2021). Importance of Stem Cell Culture Conditions for Their Derived Extracellular Vesicles Therapeutic Effect. Free Radic. Biol. Med..

[346] Cao J., Wang B., Tang T., Lv L., Ding Z., Li Z., Hu R., Wei Q., Shen A., Fu Y. (2020). Three-Dimensional Culture of MSCs Produces Exosomes with Improved Yield and Enhanced Therapeutic Efficacy for Cisplatin-Induced Acute Kidney Injury. Stem Cell Res. Ther..

[347] Patel D.B., Luthers C.R., Lerman M.J., Fisher J.P., Jay S.M. (2019). Enhanced Extracellular Vesicle Production and Ethanol-Mediated Vascularization Bioactivity via a 3D-Printed Scaffold-Perfusion Bioreactor System. Acta Biomater..

[348] Gobin J., Muradia G., Mehic J., Westwood C., Couvrette L., Stalker A., Bigelow S., Luebbert C.C., Bissonnette F.S.-D., Johnston M.J.W. (2021). Hollow-Fiber Bioreactor Production of Extracellular Vesicles from Human Bone Marrow Mesenchymal Stromal Cells Yields Nanovesicles That Mirrors the Immuno-Modulatory Antigenic Signature of the Producer Cell. Stem Cell Res. Ther..

[349] Garcia S.G., Sanroque-Muñoz M., Clos-Sansalvador M., Font-Morón M., Monguió-Tortajada M., Borràs F.E., Franquesa M. (2024). Hollow Fiber Bioreactor Allows Sustained Production of Immortalized Mesenchymal Stromal Cell-Derived Extracellular Vesicles. Extracell. Vesicles Circ. Nucleic Acids.

[350] Ludlow J.W., Buehrer B.M. (2024). Analyses and Utilization of Selectively Tuned Human Adipose-Derived Stromal/Stem Cell Exosomes. Methods Mol Biol..

[351] Gao W.-X., Sun Y.-Q., Shi J., Li C.-L., Fang S.-B., Wang D., Deng X.-Q., Wen W., Fu Q.-L. (2017). Effects of Mesenchymal Stem Cells from Human Induced Pluripotent Stem Cells on Differentiation, Maturation, and Function of Dendritic Cells. Stem Cell Res. Ther..

[352] Spitzhorn L.-S., Megges M., Wruck W., Rahman M.S., Otte J., Degistirici Ö., Meisel R., Sorg R.V., Oreffo R.O.C., Adjaye J. (2019). Human iPSC-Derived MSCs (iMSCs) from Aged Individuals Acquire a Rejuvenation Signature. Stem Cell Res. Ther..

[353] Lee J.H., Ha D.H., Go H.-K., Youn J., Kim H.-K., Jin R.C., Miller R.B., Kim D.-H., Cho B.S., Yi Y.W. (2020). Reproducible Large-Scale Isolation of Exosomes from Adipose Tissue-Derived Mesenchymal Stem/Stromal Cells and Their Application in Acute Kidney Injury. Int. J. Mol. Sci..

